# Comprehensive multi-omics analysis reveals the core role of glycerophospholipid metabolism in rheumatoid arthritis development

**DOI:** 10.1186/s13075-023-03208-2

**Published:** 2023-12-15

**Authors:** Congcong Jian, Lingli Wei, Tong Wu, Shilin Li, Tingting Wang, Jianghua Chen, Shengjia Chang, Jie Zhang, Binhan He, Jianhong Wu, Jiang Su, Jing Zhu, Min Wu, Yan Zhang, Fanxin Zeng

**Affiliations:** 1 School of Basic Medical Science, Chengdu University of Traditional Chinese Medicine, Chengdu, China; 2 Department of Clinical Research Center, Dazhou Central Hospital, Dazhou, Sichuan China; 3 Department of Rheumatology and Immunology, Dazhou Central Hospital, Dazhou, China; 4 Department of Rheumatology and Immunology, Sichuan Provincial People’s Hospital, Chengdu, China; 5 Institute of Basic Medicine and Forensic Medicine, North Sichuan Medical College, Nanchong, Sichuan China; 6 Shantou University Medical College, Shantou University, Guangdong, China; 7 Huaxi MR Research Center (HMRRC), Department of Radiology, West China Hospital of Sichuan University, Chengdu, 610041 China; 8 Lung Cancer Center of West China Hospital, Sichuan University, Chengdu, China; 9 Department of Big Data and Biomedical AI, College of Future Technology, Peking University, Beijing, 100871 China

**Keywords:** Rheumatoid arthritis, Multi-omics, New-onset RA, Chronic RA

## Abstract

**Objectives:**

Rheumatoid arthritis (RA) is a chronic autoimmune disease with complex causes and recurrent attacks that can easily develop into chronic arthritis and eventually lead to joint deformity. Our study aims to elucidate potential mechanism among control, new-onset RA (NORA) and chronic RA (CRA) with multi-omics analysis.

**Methods:**

A total of 113 RA patients and 75 controls were included in our study. Plasma and stool samples were obtained for 16S rRNA sequencing, internally transcribed spacer (ITS) sequencing and metabolomics analysis. And PBMCs were obtained for RNA sequencing. We used three models, logistic regression, least absolute shrinkage and selection operator (LASSO), and random forest, respectively, to distinguish NORA from CRA, and finally we validated model performance using an external cohort of 26 subjects.

**Results:**

Our results demonstrated intestinal flora disturbance in RA development, with significantly increased abundance of *Escherichia-Shigella* and *Proteobacteria* in NORA. We also found that the diversity was significantly reduced in CRA compared to NORA through fungi analysis. Moreover, we identified 29 differential metabolites between NORA and CRA. Pathway enrichment analysis revealed significant dysregulation of glycerophospholipid metabolism and phenylalanine metabolism pathways in RA patients. Next, we identified 40 differentially expressed genes between NORA and CRA, which acetylcholinesterase (ACHE) was the core gene and significantly enriched in glycerophospholipid metabolism pathway. Correlation analysis showed a strong negatively correlation between glycerophosphocholine and inflammatory characteristics. Additionally, we applied three approaches to develop disease classifier models that were based on plasma metabolites and gut microbiota, which effectively distinguished between new-onset and chronic RA patients in both discovery cohort and external validation cohort.

**Conclusions:**

These findings revealed that glycerophospholipid metabolism plays a crucial role in the development and progression of RA, providing new ideas for early clinical diagnosis and optimizing treatment strategies.

**Supplementary Information:**

The online version contains supplementary material available at 10.1186/s13075-023-03208-2.

## Introduction

Rheumatoid arthritis (RA) is a chronic, systemic autoimmune disease, characterized by symmetrical synovial inflammation and eventual involvement of other organ systems [1–3]. According to epidemiological surveys, the global prevalence of RA is 0.2–1.0%, and nearly 5 million people in China suffer from RA, with a prevalence of 0.28–0.41% [4]. The pathogenesis of RA is not fully understood, and its highly specific, inherited, and environmental factors combine to influence the onset and progression of the disease [5–7]. Symptoms of new-onset RA (NORA) are mainly characterized by high disease activity, joint inflammation, and pain. Most patients have delayed treatment due to ineffective treatment regimens or poor compliance, and as the disease progresses and the inflammatory state worsens, the joints and articular cartilage were destroyed, eventually evolving into chronic rheumatoid arthritis (CRA) with a range of extra-articular damages [8, 9]. Therefore, early diagnosis and early treatment of RA can effectively prevent disease progression, joint damage, and destruction of other organ systems in most patients.

Among the many influential factors, intestinal flora is considered to be an important trigger for immune system abnormalities in RA [10]. Previous studies showed that a decrease in the composition and diversity of the intestinal flora in RA patients, with an increase in the abundance of *Klebsiella*, *Escherichia*, and a decrease in the abundance of *Megamonas* and *Enterococcus*, and an expansion of *Prevotella* associated with an increased susceptibility to arthritis, suggesting that the development of RA is closely associated with dysbiosis of the intestinal flora [11, 12]. In recent years, with the development of metabolomics technology, more and more evidences revealed that patients with RA have significant changes in plasma metabolites and metabolic pathways, such as lipid metabolism and amino acid metabolism [11, 13]. Transcriptomic analysis is widely used in the field of RA research. Gene expression profiles of peripheral blood mononuclear cells (PBMCs) could reveal the pathological process and pathogenesis of RA involving immune cells and play an important role in predicting response to drug therapy, screening for key differential genes and explaining the pathogenesis of RA [14, 15]. A study had demonstrated that HLA-DRB1 was a susceptibility gene that triggers RA, affecting disease activity and treatment response [16]. At present, multi-omics combined analysis is broadly applied in the research field, which can explore the interactions and potential links between gut microbes and metabolites, and elucidate the pathogenesis of diseases as a result of the combined influence of multiple factors. However, multi-omics researches are less well studied in explaining the pathogenesis of RA development, and it is essential to use multi-omics approaches to gain a comprehensive understanding of the pathogenesis of RA.

In our study, we combine gut flora, plasma metabolism, and transcriptome analysis to explore the changes and potential relationships between control, NORA, and CRA patients, to elucidate the interactions between gut microbes, plasma metabolites, and genes, and to reveal the pathogenesis of RA with the joint influence of multiple factors, providing a new perspective and research direction for early clinical diagnosis and precise treatment.

## Materials and methods

### Participant recruitment

The study population consisted of RA patients aged > 18 years from the Department of Rheumatology and Immunology, and control from the medical examination center, Dazhou Central Hospital. NORA included those with less than 6 months of disease and who have never used antirheumatic drugs, CRA with more than 6 months of disease who have used traditional antirheumatic drugs (methotrexate, leflunomide, and hydroxychloroquine), and were accompanied by the use of non-steroidal anti-inflammatory drugs (NSAIDs) and glucocorticoids. Patients were diagnosed with rheumatoid arthritis in this study according to the 2010 American College of Rheumatology (ACR) and European League of Rheumatology (EULAR) criteria [17]. Clinical data from RA patients were recorded, including rheumatoid factor (RF), erythrocyte sedimentation rate (ESR), C-reactive protein (CRP), 28 tender joints count (TJC28), 28 swollen joints count (SJC28), disease activity score of 28 joints (DAS28), and interleukin-6 (IL-6).

### Study design

**Fig. 1:**
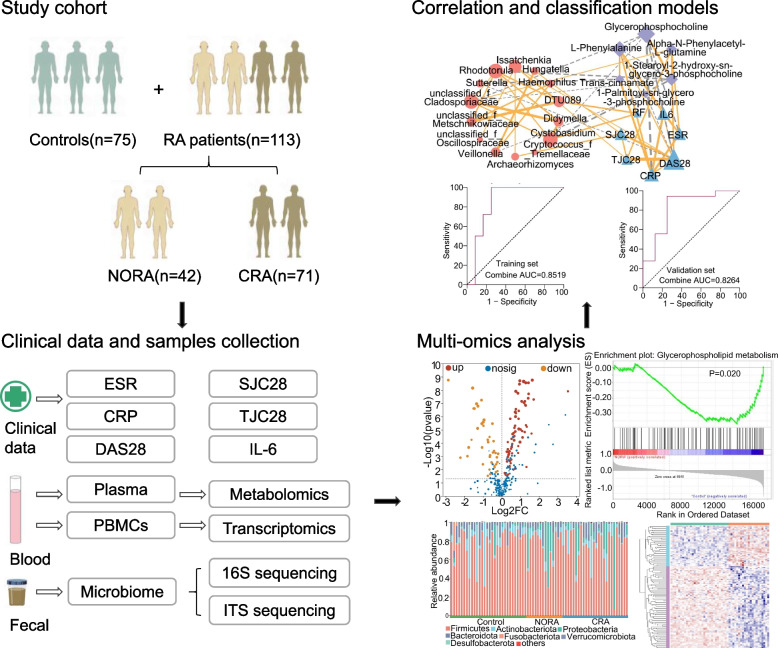
Overview of the study design. NORA, new-onset rheumatoid arthritis; CRA, chronic rheumatoid arthritis; ESR, erythrocyte sedimentation rate; CRP, C-reactive protein; DAS28, disease activity score; SJC28, number of swollen joints; TJC28, number of tender joints; IL-6, interleukin-6; PBMCs, peripheral blood mononuclear cells

Our study included 188 subjects (NORA = 42, CRA = 71, control = 75), stool and plasma samples were collected for 16S, ITS sequencing and metabolomics analysis respectively, to identify differential flora and differential metabolites between control, NORA, and CRA. PBMCs were collected for transcriptome sequencing to identify differentially expressed genes among three groups, and finally, a combined multi-omics analysis and correlation and classification analysis was performed to establish classification models, and we use an external cohort to verify the performance of the model details as described in Fig. 1.

### Stool sample DNA extraction and Illumina sequencing

Total DNA extraction from stool samples of the subjects were performed according to the instructions. 16S rRNA sequencing V3–V4 variable region PCR amplification primers were 338F (5′-ACTCCTACGGGAGGCAGCAG-3′) and 806R (5′-GGACTACHVGGGTWTCTAAT-3′), and ITS amplification primers were ITS1F (5′-CTTGGTCATTTAGAGGAAGTAA-3′) and ITS2R (5′-GCTGCGTTCATCGATGC-3′), and sequenced using Illumina Miseq PE300 platform (Majorbio Bio-Pharm Technology Co. Ltd. (Shanghai, China)).

### Microbiomics data processing and analysis

The raw data we obtained for sequencing were firstly quality controlled and spliced by fastp and FLASH software, and secondly, the sequences were processed for noise reduction using the DADA2 plug-in to obtain amplicon sequence variants (ASVs). We sampled the amplicon ASVs by minimum number of sequences, and subsequently we performed gut microbial community composition analysis, alpha diversity, and beta diversity analysis on the Majorbio cloud platform (https://cloud.majorbio.com). In alpha diversity analyses, mothur-1.30 analyses were used with the rank-sum test method, and the Tukey–Kramer method was used for the calculation of 95% confidence intervals; in beta diversity analyses, PCoA analyses were performed using R software analyses (R-3.3.1 ( vegan)) Bray–Curtis distance algorithm, Adonis for between-group difference tests; python-2.7 was used for species composition analysis; in differential species analysis, R-3.3.1 (stat) was used, Wilcoxon rank-sum two-tailed test, and 95% confidence intervals were computed using the bootstrap method. Detailed depictions of specific analysis methods are described in Supplementary Material 1.

### Non-targeted metabolomics analysis

We collected plasma samples from subjects for liquid chromatography-tandem mass spectrometry (LC–MS/MS) analysis through a bioassay company (Majorbio Bio-Pharm Technology Co. Ltd. Shanghai, China). We obtained raw metabolic data which were first pre-processed using the metabolomics software Progenesis QI (Waters Corporation, Milford, USA), removing missing values > 80% in each group, filling missing values with the minimum values, and removing metabolites with relative standard deviations (RSD) > 30% in QC samples. Subsequently, the mass spectral information of the metabolites was matched with the metabolic public databases HMDB (http://www.hmdb.ca/) and Metlin (https://metlin.scripps.edu/) to obtain the final metabolite expression profiles for subsequent analysis of the results. The results of differential metabolite analysis and Kyoto Encyclopedia of Genes and Genomes (KEGG) enrichment analyses were carried out on the online platform of Majorbio (https://cloud.majorbio.com), in which the differential metabolite analysis Metabolites with VIP > 1 and *p* < 0.05 were identified as significant differential metabolites using the variable weight values (VIP) obtained from the orthogonal partial least squares discriminant analysis (OPLS-DA) model and Wilcox test *p*-value. In enrichment analysis, KEGG enrichment pathway used topology methods, the Python package scipy.stats, and the pathways involved in the differential metabolites were obtained from the metabolic pathway annotations in the KEGG database (https://www.kegg.jp/kegg/pathway.html). A detailed description of the metabolomics methods can be found in Supplementary Material 2.

### Transcriptomic sequencing and data analysis

We collected PBMCs from subjects for transcriptomic sequencing and total RNA was extracted by the company’s (Novogene, Beijing) standard extraction method and quality control and RNA integrity assays were performed using an Agilent 2100 Bioanalyzer. Libraries were prepared using the NEBNext® Ultra™ RNA library Prep Kit for Illumina®, and libraries were sequenced using the Illumina NovaSeq 6000 after passing library assays. Finally, reads were generated using the HISAT2 (v2.0.5) reference gene library and genetically matched to the reads, resulting in final data for subsequent bioinformatics analysis. We performed differentially expressed genes screening, KEGG and Gene Ontology (GO) enrichment analyses on the Novogene online platform (https://magic.novogene.com/). In the differentially expressed gene analysis, we first normalized the read count expression matrix, followed by statistical analysis and mapping using the R (Version 3.0.3) ggplot2 package. Simultaneously, we used clusterProfiler software to perform GO functional enrichment analysis and KEGG pathway enrichment analysis on the differential gene sets. In addition, we used Gene Set Enrichment Analysis (GSEA_4.3.2) for pathway enrichment analysis and also employed STRING for protein–protein interaction network analysis.

### Multi-omics integration and models establishment

We selected differential gut flora (bacteria and fungi) and differential metabolites significantly in glycerophospholipid metabolism and phenylalanine metabolism pathways between NORA and CRA for Spearman correlation analysis, and then visualized them on Cytoscape software. Moreover, we applied logistic regression, LASSO and random forest for screening features to build the classification models, as well as an independent cohort to validate the performance of the models.

### Statistical analysis

In the course of data processing, we also used SPSS Statistics (V.25) and GraphPad Prism (v8.0.2) for data statistical analysis.

## Results

### Clinical differences in RA patients

In our study, we found that SJC28 and the level of IL-6 were higher in NORA than in CRA patients and were apparently significant. We also noticed that CRP, TJC28, and DAS28 were higher in NORA compared to CRA, although there was no difference between them. Similarly, the levels of ESR and RF were not different between two groups. In the validation cohort, we observed a significant difference in RF level between the two groups, as detailed in Supplementary Table 1.

### Dysregulation of metabolites and metabolism pathways in the plasma metabolic profile of RA patients

**Fig. 2:**
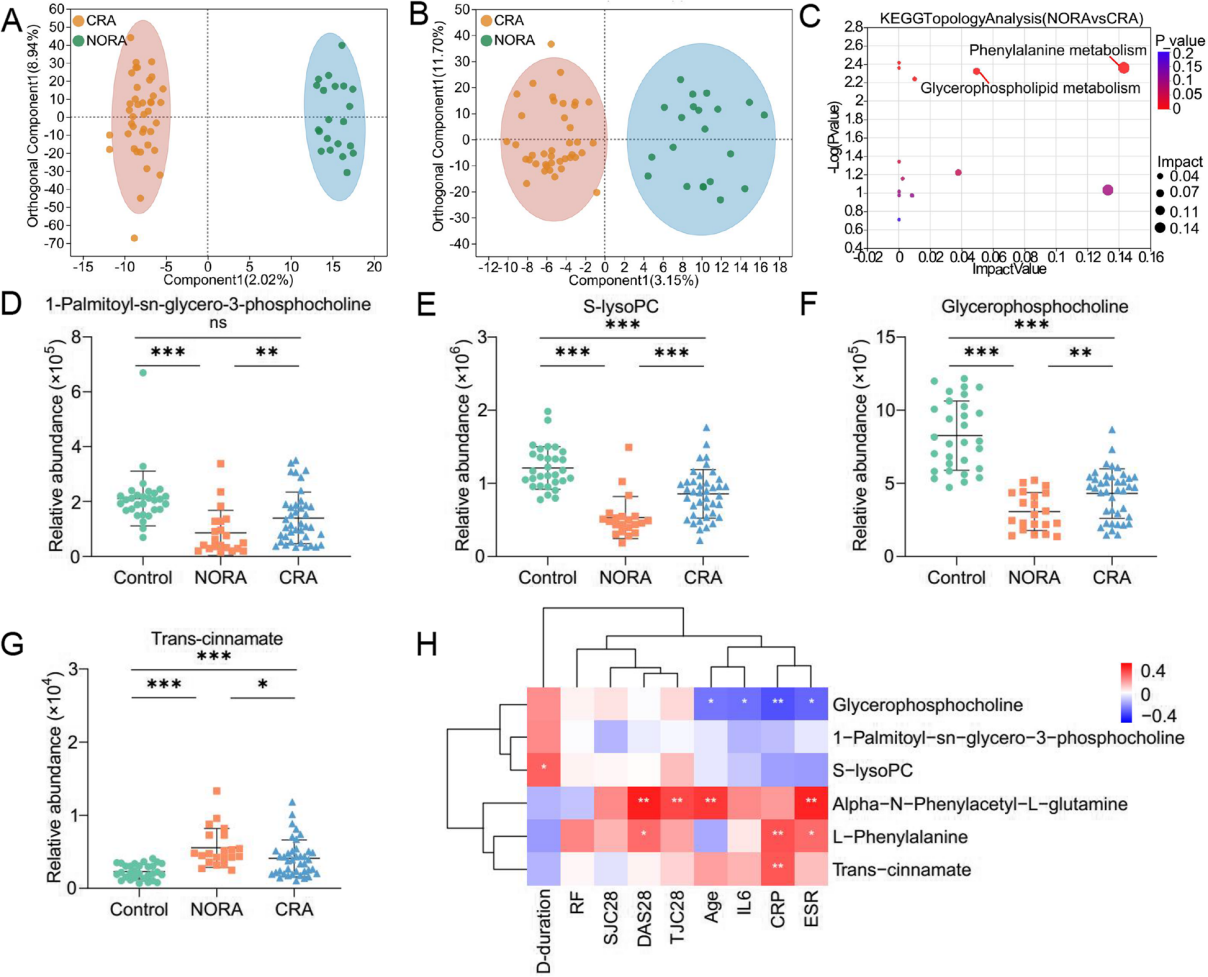
Identification of differential metabolites in plasma metabolic profile between NORA and CRA patients.** A**, **B** OPLA-DA analysis showed 29 differential metabolites between NORA and CRA in anionic and cationic patterns, respectively. **C** The bubble diagram showed a significant enrichment of differential metabolites between NORA and CRA in the glycerophospholipid metabolism and phenylalanine metabolism pathways, including 1-Palmitoyl-sn-glycero-3-phosphocholine, S-lysoPC, glycerophosphocholine, trans-cinnamate, L-Phenylalanine, and alpha-N-Phenylacetyl-L-glutamine. **D**–**G** Scatter plots demonstrated the relative abundance of 4 differential metabolites on glycerophospholipid metabolism and phenylalanine metabolism pathways between controls, NORA, and CRA. **H** Correlation heatmap showed the interrelationship between 6 differential metabolites and clinical characteristics. (S-lysoPC: 1-Stearoyl-2-hydroxy-sn-glycero-3-phosphocholine; D-duration: disease duration)

To identify differential metabolites between control, NORA and CRA groups, we performed non-target metabolomics analysis of three groups. We found 111 differential metabolites between NORA and control, of which 66 were anionic and 45 were cationic metabolites. OPLS-DA demonstrated a clear separation of differential metabolites between two groups in the anionic and cationic modes (Supplementary Figure 1A,B). A volcano plot showed 111 differentially significant metabolites, with 70 upregulated and 41 downregulated significant metabolites (Supplementary Figure 1C). Meanwhile, we identified 110 differential metabolites between CRA and control, and OPLS-DA plots illustrated a clear distinction between differential metabolites in anionic and cationic modes, respectively (Supplementary Figure 1D-E), and volcano demonstrated significant upregulation of 41 metabolites and downregulation of 69 metabolites (Supplementary Figure 1F). Furthermore, we also identified 29 differentiated metabolites between NORA and CRA patients, and OPLS-DA plots demonstrated an apparent differentiation between two groups in terms of differential metabolites in anionic and cationic modes (Fig. 2A,B). Volcano plot illustrated that 10 differential metabolites were significantly upregulated and 19 differential metabolites were significantly downregulated (Supplementary Figure 1G). To observe the clustering of differential metabolites between groups, the heat maps showed 111, 110, and 29 differential metabolites clearly distinguished between the three groups, respectively (Supplementary Figure 1H-J).

Afterwards, we performed KEGG enrichment analysis of differential metabolites and found 6 metabolic pathways were altered in NORA and 9 metabolic pathways were changed in CRA patients compared to control, with significant deregulation of glycerophospholipid metabolism, histidine metabolism, citrate cycle (TCA cycle), and glycine, serine, and threonine metabolism pathways common among them (Supplementary Figure 1K-L). We also detected glycerophospholipid metabolism and phenylalanine metabolism pathways were significantly disordered between NORA and CRA patients, including 6 differentially significant metabolites (1-Palmitoyl-sn-glycero-3-phosphocholine, 1-Stearoyl-2-hydroxy-sn-glycero-3- phosphocholine (S-lysoPC), glycerophosphocholine, trans-cinnamate, L-Phenylalanine and alpha-N-Phenylacetyl-L-glutamine) (Fig. 2C–G, Supplementary Figure 1M-N, Supplementary Table 2), and 6 differential metabolites were also significantly differentiated between NORA versus control and CRA versus control. More importantly, we found that the expression levels of the first three metabolites were higher in controls than in RA patients, while the last three metabolites were higher in RA patients than in controls. Therefore, we performed correlation analysis of 6 metabolites, and the heatmap demonstrated a significant positively correlation between trans-cinnamate and L-Phenylalanine, and a strong positively association among 1-Palmitoyl-sn-glycero-3-phosphocholine, S-lysoPC, and glycerophosphocholine. While a significant negatively correlation between glycerophosphocholine and trans-cinnamate (Supplementary Figure 1O). Simultaneously, we found strong correlations between six differential metabolites and clinical characteristics, with significant negative correlations between glycerophosphocholine and ESR, CRP, IL-6, and age, positive correlations between S-lysoPC and disease duration. The heat map also exhibited a significant positive correlation between DAS28, CRP, and L-Phenylalanine (Fig. 2H).

These results suggested significant alterations in plasma metabolic profile of RA patients, with disturbed amino acid metabolism and lipid metabolic pathways, and glycerophospholipid metabolism pathway appeared to play an important role in the progression of RA disease.

### An apparently increased abundance of Escherichia-Shigella and decreased Bifidobacterium in RA patients

**Fig. 3:**
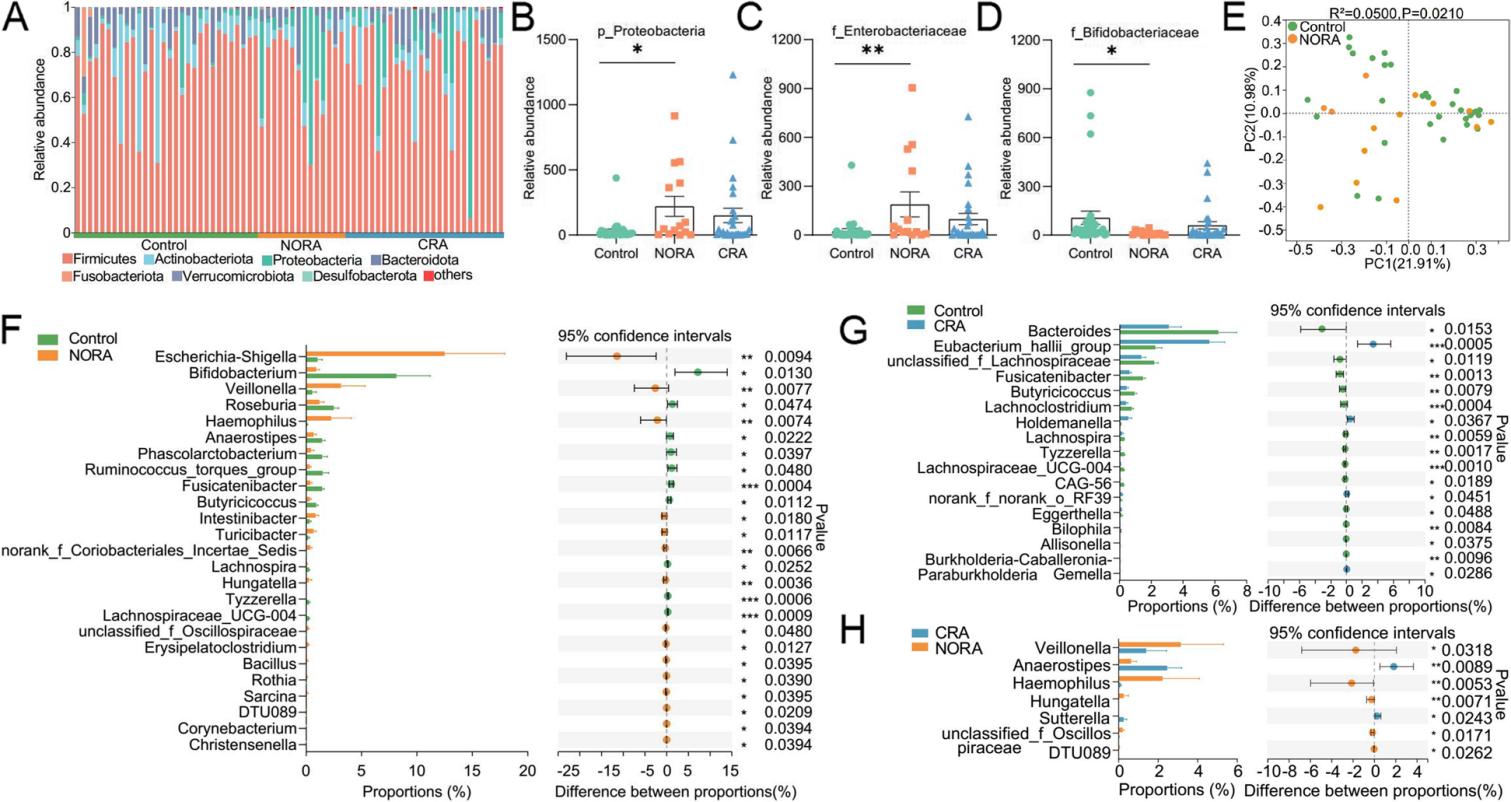
Alterations in the structural composition and diversity of the intestinal bacterial community.** A** Stacked bar graph showed the community composition at the phylum level among control, NORA, and CRA. **B** Bar plots displayed the relative abundance expression of *P_Proteobacteria* in three groups, indicating a significant difference in abundance between new-onset RA and control. **C**, **D** The bar graphs showed the relative abundance of *f_Enterobacteriaceae* and *f_Bifidobacteriaceae* among the three groups, respectively. **E** PCoA revealed differential community structure between NORA and control in the beta diversity analysis. Adonis between-group difference test using Bray–Curtis distance algorithm, analyzed by number of 999 substitutions. **F**–**H** Using the Wilcoxon rank-sum test, we identified 26 differential genera between NORA and control, 17 differential genera between CRA and control, and 7 differential genera between NORA and CRA. Values represented mean and standard error. Control (*n* = 30), NORA (*n* = 14), CRA (*n* = 26)

To investigate the community structure of intestinal bacteria among control, NORA, and CRA patients, we performed 16S rRNA sequencing on stool samples from subjects. We found a reduced number of gut bacteria in NORA compared to control, although there was no significant difference between them, and the Venn diagram showed a total of 133 species between the three groups (Supplementary Figure 2A). In community composition analysis, the relative abundance of the *Proteobacteria* was significantly increased at the phylum level in NORA compared to control, while the abundance of *Firmicutes*, *Bacteroidota*, and *Actinobacteriota* did not differ between the three groups (Fig. 3A,B, Supplementary Figure 2B-D). At the family level, stacked bar chart showed the relative abundance of each family in the three groups in different samples, and we found that *Lachnospiraceae* was the dominant species in three groups (Supplementary Figure 2E). The relative abundance of *Pasteurellaceae* was significantly increased in NORA compared to control and CRA groups (Supplementary Figure 2F). Moreover, we found an apparent increase in the abundance of *Enterobacteriaceae* and decrease in the abundance of *Bifidobacteriaceae* and *Acidaminococcaceae* in NORA patients (Fig. 3C,D, Supplementary Figure 2G). We also focused on a reduced abundance of *Bacteroidaceae* in CRA, while *Prevotellaceae* and *Lactobacillaceae* did not differ between the three groups (Supplementary Figure 2H-J). In alpha diversity analysis, we discovered no significant differences in community richness and diversity between control, NORA, and CRA patients (Supplementary Figure 3A-F). In beta diversity based on Bray–Curtis distance, principal co-ordinates analysis (PCoA) showed that NORA could be distinguished from control (*P* = 0.021), whereas no distinction could be observed between CRA and control (*P* = 0.055) and NORA compared with CRA (*P* = 0.535) (Fig. 3E, Supplementary Figure 3G-H). Applying the Wilcoxon rank-sum test, we found 26 differential genera at genus level between controls and NORA, with *Escherichia-Shigella* and *Veillonella* significantly increasing in abundance and *Bifidobacterium* decreasing in NORA (Fig. 3F). Meanwhile, we identified 17 differential genera between CRA and controls, with a dramatically increased abundance of *Eubacterium_hallii_group* in CRA, and we also identified 7 differential genera between NORA and CRA, with a significant decrease in abundance of *Veillonella* and *Haemophilus*, and *Anaerostipes* significantly increased in CRA (Fig. 3G–H). LDA demonstrated the importance of species from phylum to genus level among control, NORA, and CRA groups, we found that *f_Bifidobacteriaceae*, *g_Bifidobacterium*, *o_Bifidobacteriales*, and *c_Actinobacteria* were dominant in control, while *p_Proteobacteria*, *g_Escherichia-Shigella* and *g_Veillonella* were predominant in NORA. Compared to CRA, *g_Bacteroides* and *f_Bacteroidaceae* were the predominant genera in control, and LDA analysis demonstrated the significance of *f_Pasteurellaceae* and *g_Anaerostipes* in both groups in NORA versus CRA (Supplementary Figure 3I-K).

These results indicated a significant variation in the abundance of gut bacteria from phylum to genus level between the three groups. It reflected the disruption of the intestinal microecosystem and dysbiosis of the intestinal flora during the disease state, which exacerbated the inflammatory response and contributed to the progression of RA.

### Altered diversity of intestinal fungi among control, NORA, and CRA groups

**Fig. 4:**
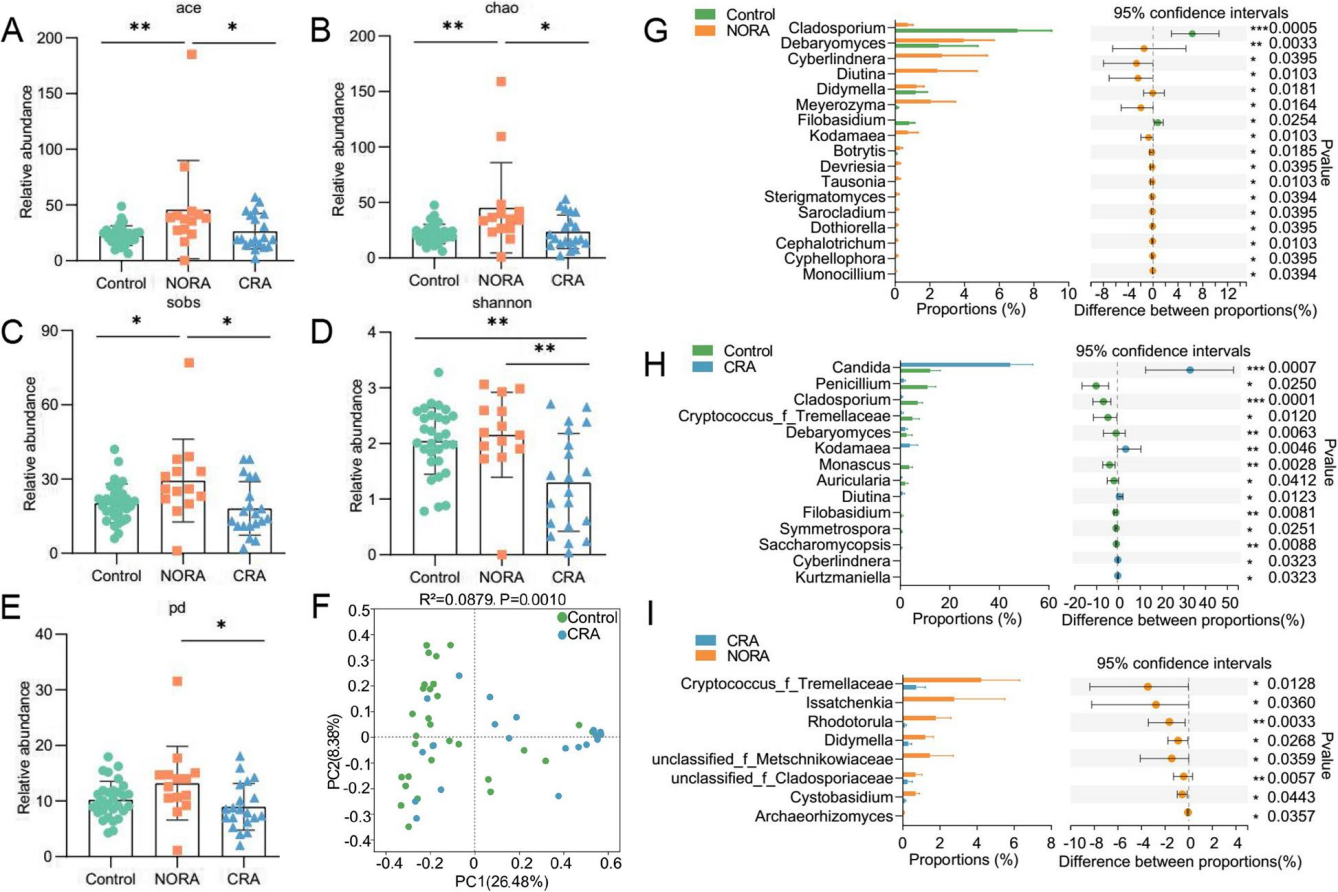
Changed intestinal fungal community composition and reduced diversity in CRA patients.** A**–**E** The bar graphs displayed the relative abundance of ace, chao, sob, shannon, and pd indexes between the three groups, respectively, with significantly lower community richness and diversity in CRA. Kruskal–Wallis rank-sum test for three group comparisons, with the error bars representing the standard deviation. **F** PCoA demonstrated significant differences in community structure between CRA and control. Adonis between-group difference test using Bray–Curtis distance algorithm, analyzed by number of 999 substitutions. **G**–**I** Using the Wilcoxon rank-sum test, we identified 17 differential genera between NORA and control, 14 differential genera between CRA and control, and 8 differential genera between NORA and CRA. Values represented mean and standard error. Control (*n* = 30), NORA (*n* = 14), CRA (*n* = 20)

To observe the alterations in intestinal fungal diversity and composition between control, NORA, and CRA groups, we performed ITS sequencing on stool samples from subjects. Venn diagram showed a total of 64 species across three groups and 14 species between NORA and CRA patients (Supplementary Figure 4A). In alpha diversity analysis, we found a measurable increase in community richness in Ace, Chao, and Sobs indexes for NORA compared to control and CRA. The diversity of Shannon and pd indexes were obvious reduced in CRA compared with NORA (Fig. 4A–E). We found that the abundance of *Aspergillaceae* was significantly higher in control than in RA patients as the dominant genus, while *Saccharomycetales_fam_Incertae_sedis* was the dominant genus in RA at family level with community composition analysis (Supplementary Figure 4B). The abundance of *Cladosporiaceae* was significantly reduced in RA patients compared to control, and the abundance of *Phaffomycetaceae*, *Debaryomycetaceae*, and *Didymellaceae* was increased in NORA (Supplementary Figure 4C-F). PCoA analysis demonstrated that control and CRA could be distinguished from each other, whereas NORA could not be distinctly separated from control and NORA from CRA (Fig. 4F, Supplementary Figure 3G-H). Subsequently, using the Wilcoxon rank-sum test, we identified 17 differential genera between control and NORA, and 14 genera differed in control and CRA groups, with an apparent decrease of *Cladosporium* in RA. *Candida* was significantly increased in CRA compared to control. In addition, we also found 8 differential genera between NORA and CRA (Fig. 4G–I).

Taken together, these data suggested that dysbiosis of intestinal fungi was closely related to the progression of RA.

### Gene expression profiles were significantly dysregulated between control, NORA, and CRA patients

**Fig. 5:**
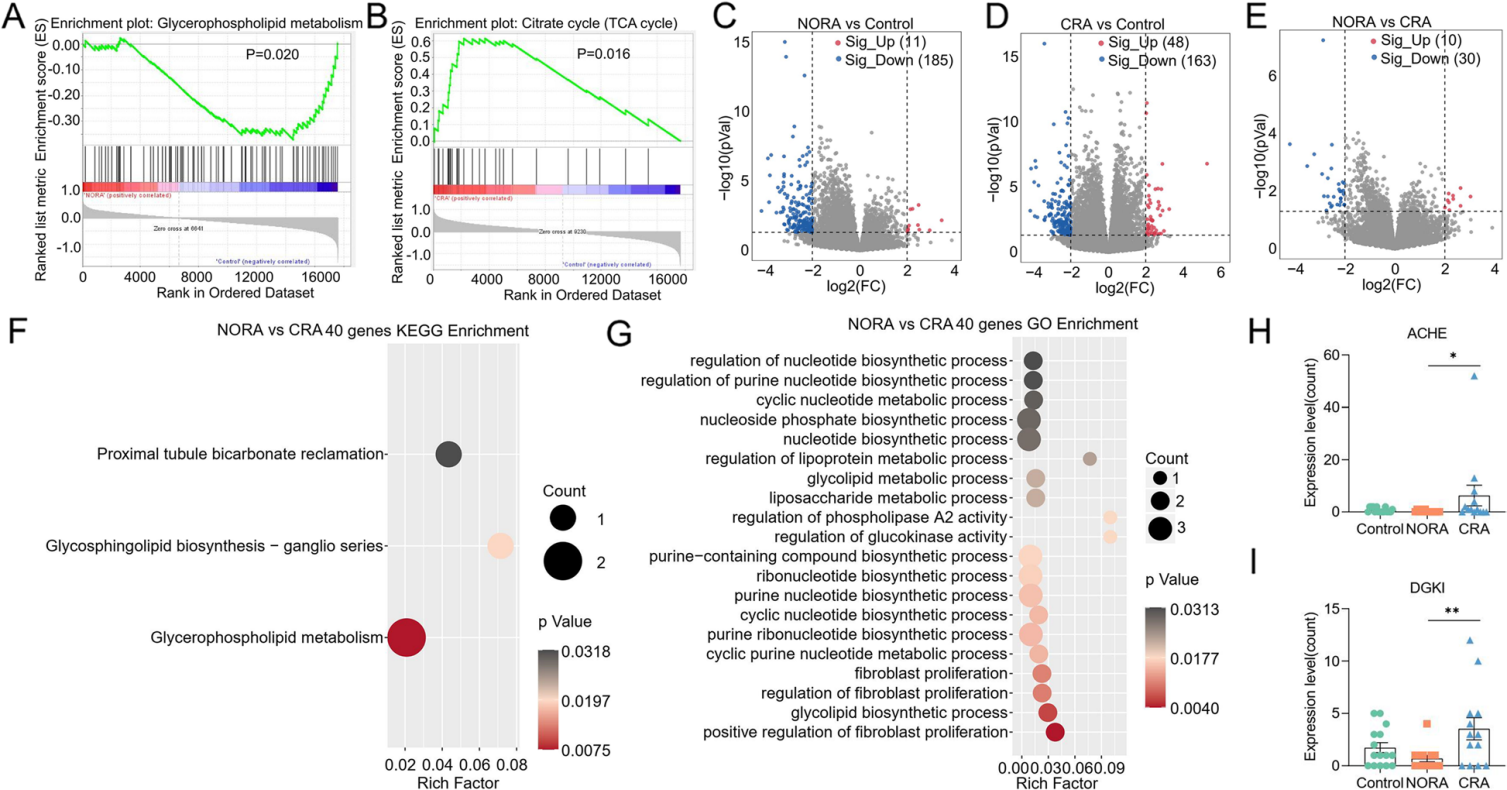
Differences in gene expression profile and enrichment analysis between NORA and CRA groups.** A** GSEA (Gene Set Enrichment Analysis) enrichment analysis demonstrated that glycerophospholipid metabolism was significantly enriched in the control compared to NORA group. **B** GSEA enrichment analysis demonstrated a significant enrichment of citrate cycle (TCA) cycle in CRA compared to control. **C** Volcano plot showed differentially expressed genes between NORA and control, with 11 upregulated and 185 downregulated genes. **D** Volcano plot showed 211 differentially expressed genes between CRA and control, with 48 upregulated and 163 under-regulated genes. **E** Volcano plot showed 40 differentially expressed genes between NORA and CRA, with 10 were upregulated and 30 were downregulated. **F**, **G** Bubble plots illustrated the results of KEGG and GO enrichment analysis for 40 differentially expressed genes, respectively. **H**, **I** The bar graphs demonstrated the expression of ACHE and DGKI genes in the glycerophospholipid metabolism pathway

To further explore the characteristics of gene expression profiles in control, NORA, and CRA, we performed RNA sequencing of PBMCs from subjects. Among NORA versus controls, we selected protein-coding genes for GSEA, of which 9 gene sets were significantly enriched in control, including glycerophospholipid metabolism, glycosylphosphatidylinositol gpi anchor biosynthesis, calcium signaling pathway, taste transduction, neuroactive ligand receptor interaction, RNA polymerase, arachidonic acid metabolism, hedgehog signaling pathway, and basal cell carcinoma (Fig. 5A, Supplementary Figure 5A-H, Supplementary Table 3). Furthermore, of the protein-coding genes we screened in CRA versus control, GSEA analysis showed 3 gene sets enriched in control and 16 gene sets enriched in CRA, like citrate cycle (TCA cycle) (Fig. 5B, Supplementary Table 4). Subsequently, we identified 196 differentially expressed genes between NORA and control, a volcano plot showed 11 significantly upregulated and 185 significantly downregulated genes (|FC|> 4, *P* < 0.05). Between CRA and control, we found 211 differentially expressed genes and volcano displayed 48 genes significantly upregulated and 163 downregulated (|FC|> 4, *P* < 0.05). Also, we identified 40 differential genes between NORA and CRA, and the volcano plot demonstrated significant upregulation of 10 genes and downregulation of 30 genes (|FC|> 4, *P* < 0.05) (Fig. 5C–E). Moreover, we performed KEGG enrichment analysis on 196 genes and identified 3 pathways that were significantly dysregulated. We also identified 89 pathways significantly disordered by GO enrichment analysis, and the bubble demonstrated 22 of them, and we noticed that the differential genes were mainly enriched in metabolic process (Supplementary Figure 5I-J, Supplementary Table 5). Based on 211 differentially expressed genes between CRA and control, we identified 23 pathways significantly enriched using KEGG enrichment analysis. In total, 253 pathways were discovered to be apparently altered in GO analysis, with bubble showing 24 of them (Supplementary Figure 5K-L, Supplementary Table 6). More importantly, we performed KEGG enrichment analysis of 40 differentially expressed genes between NORA and CRA patients. We identified 3 pathways significantly dysregulated, including glycerophospholipid metabolism, glycosphingolipid biosynthesis—ganglio series and proximal tubule bicarbonate reclamation. Using GO enrichment analysis, we identified 128 pathways of biological process significantly dysregulated, and bubble plot showed 20 of them, mainly enriched in biosynthetic and metabolic processes (Fig. 5F–G, Supplementary Table 7). Among the -128 GO terms, we found significant dysregulation of the Wnt signaling pathway, the G protein-coupled receptor signaling pathway, and the negative regulation of cAMP-mediated signaling, these pathways were closely associated with the immune system (Supplementary Table 7). Furthermore, we found the expressions of ACHE and DGKI were significantly increased in CRA compared to NORA patients in glycerophospholipid metabolism pathway, which may be associated with dysregulated lipid metabolism (Fig. 5H, I).

These outcomes indicated a significantly dysregulated gene expression profile in RA patients and a predominant enrichment in the glycerophospholipid metabolism pathway.

### Multi-omics combined analysis and ROC classification models establishment

**Fig. 6:**
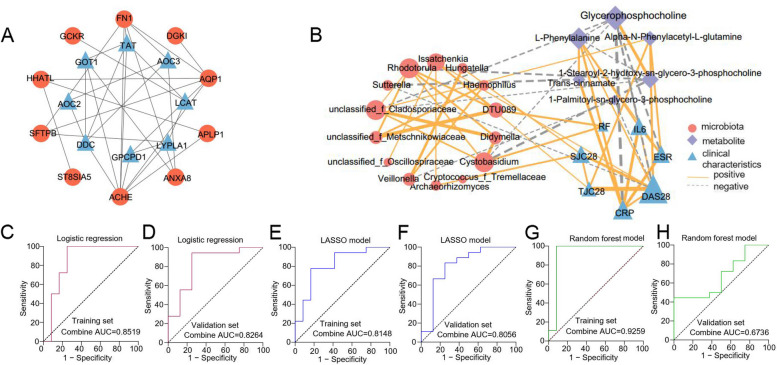
Multi-omics combined analysis and ROC classification models establishment.** A** Protein–protein interaction network analysis illustrated the interconnections between 12 differentially expressed genes and proteins on the glycerophospholipid metabolism and phenylalanine metabolism pathways between NORA and CRA. Red represented 12 of the 40 differentially expressed genes between NORA and CRA, and blue represented the proteins on the glycerophospholipid metabolism and phenylalanine metabolism pathways. **B** Correlation heatmap and network revealed the interrelationship between differential bacteria, differential fungi, differential metabolites, and clinical inflammatory indicators, showing a strong correlation between flora, metabolites, and inflammatory features. Red represented the differential flora, purple represented the differential metabolites, and blue represented the clinical inflammatory features. The size of the graph represented degree, the thick line of the line represented the correlation, the solid line represented the positive correlation, and the dashed line represented the negative correlation. **C**, **D** ROC analysis demonstrated a combined AUC of 0.8519 and 0.8264 for training set and validation set of 4 features selected by logistic regression, respectively. **E**, **F** ROC analysis demonstrated a combined AUC of 0.8148 and 0.8056 for training set and validation set of 2 features selected by LASSO. **G**, **H** ROC analysis demonstrated a combined AUC of 0.9259 and 0.6736 for training set and validation set of 6 features selected by random forest. ROC: receiver operating characteristic

To further explore the potential interrelationship between differential metabolites and genes, we focused on the interconnection between proteins in the glycerophospholipid metabolism and phenylalanine metabolism pathways enriched by 29 differential metabolites and 12 genes in the differential gene enrichment analysis. Protein–protein interaction network analysis demonstrated that acetylcholinesterase (ACHE), fibronectin 1 (FN1), and aquaporin 1 (AQP1) were core genes, and we found that ACHE was interlinked with lysophospholipase I (LYPLA1), AQP1 and FN1, and lecithin-cholesterol acyltransferase (LCAT) was interlinked with LYPLA1, glucokinase regulator (GCKR) and tyrosine aminotransferase (TAT). The ACHE gene was enriched in the glycerophospholipid metabolism pathway, and the downstream metabolites of LCAT and LYPLA1 proteins were the differential metabolites 1-Palmitoyl-sn-glycero-3-phosphocholine, 1-Stearoyl-2-hydroxy-sn-glycero-3- phosphocholine, and glycerophosphocholine, suggesting that the LCAT and LYPLA1 proteins interact with the ACHE gene, resulting in altered metabolites in the glycerophospholipid metabolic pathway (Fig. 6A). Correlation heatmap and network demonstrated strong correlation between differential flora, differential metabolites, and clinical inflammatory indicators, and we found that CRP, DAS28, ESR, and IL-6 were significantly negatively correlated with glycerophosphocholine, whereas positively correlated with the level of L-Phenylalanine. Trans-cinnamate showed a significantly positive correlation with CRP and negative correlation with *Sutterella*. DAS28, ESR, and TJC28 showed a significant positively correlation with alpha-N-Phenylacetyl-L-glutamine. *Veillonella* was negatively correlated with 1-Palmitoyl-sn-glycero-3-phosphocholine, while positively correlated with *Hungatella* and *Haemophilus* genera (Fig. 6B, Supplementary Figure 6A).

To identify crucial biomarkers to illustrate the differences between NORA and CRA, we selected 10 candidate markers with an area under the curve greater than 0.7 based on 7 differential bacteria, 8 differential fungi, and 6 differential metabolites (Supplementary Figure 6A-C). Subsequently, we applied logistic regression, LASSO, and random forest algorithms to identify essential features to build classification models to distinguish NORA from CRA patients, respectively, and we evaluated and validated the performance of the three models using an external cohort of 26 subjects. We filtered 4 key features (1-Stearoyl-2-hydroxy-sn-glycero-3-phosphocholine, glycerophosphocholine, *g_Rhodotorula*, *g_Cystobasidium*) by logistic regression with an area under the curve (AUC) of 0.8519 and 0.8264 for the validation cohort. Meanwhile, the LASSO machine learning algorithm selected *g_Cystobasidium* and glycerophosphocholine with AUCs of 0.8148 and 0.8056 for the training and validation sets, respectively. The random forest algorithm screened 6 key features with an AUC of 0.9259 for the training set, while the AUC was 0.6736 for the validation cohort (Fig. 6C–H, Supplementary Figure 7D-F). These results demonstrated that logistic regression and LASSO can distinguish between NORA and CRA patients with good performance.

The data suggested a potential relationship between intestinal flora, metabolites, and clinical inflammatory indicators, which play an important role in the progression of RA.

## Discussions

Our findings explain the dysregulation in plasma metabolic profiles, gut flora, and gene expression profiles between the control, NORA, and CRA, revealing that the pathogenesis of RA involves multi-factor interaction and regulation. We found that the differential metabolites between NORA and CRA are significantly enriched in glycerophospholipid metabolism and phenylalanine metabolism pathways. Meanwhile, we also found that the dysbiosis in the abundance and diversity of intestinal flora in RA patients. Additionally, we found that the differential genes are enriched in lipid metabolism, amino acid metabolism, biosynthesis, and metabolic processes. Lastly, correlation analysis suggested a strong association between flora, metabolites, genes, and clinical features in RA development.

Currently, a growing body of evidence suggests that the occurrence and development of RA are intimately tied to intestinal flora dysbiosis, and experimental studies have shown that intestinal dysbiosis triggers arthritis in mouse models [18–21]. Previous studies have confirmed that *Prevotella* is significantly increased in the intestine of early-diagnosed RA patients and activated the immune system and immune response, suggesting that *Prevotella* alterations appear to be a crucial factor in the pathogenesis of RA [12, 22, 23]. The composition and diversity of intestinal flora were significantly reduced in RA patients, along with a decrease in the abundance of beneficial bacteria and an increase in the abundance of harmful bacteria, and it has been proved that probiotics can slow the progression of RA and lower levels of inflammatory factors [24]. According to our findings, NORA patients had higher abundance of the bacteria *Escherichia-Shigella* and *Veillonella* and lower abundance of *Bifidobacterium*, which accelerated the progression of RA, in agreement with previous findings [12, 25, 26]. However, some studies on Chinese RA patients have found a rise in *Lactobacillus* abundance during acute phase of RA, suggesting that the role of probiotics in the development of RA remains unclear [27, 28]. We also observed that the abundance of *Veillonella* was reduced and differential in patients with CRA when compared with NORA, indicating that the inflammatory state may influence the change in the abundance of the flora. *Proteobacteria* were more prevalent and *Firmicutes* and *Bacteroidota* were less prevalent in RA patients, which was in line with previous research findings [11, 25]. The gut bacterial community structure was similar between NORA and CRA patients, but β diversity revealed a substantial divergence between control and NORA patients, indicating that intestinal bacteria were distinct between RA and control. Intestinal fungi play an important role in the development of RA, and in our study, we discovered a significantly decreased abundance and diversity in patients with CRA compared to NORA, as well as significantly increased abundance of *Candida*. These findings are consistent with those of Sokol et al. in patients with inflammatory bowel disease, which suggests that altered abundance of *Candida* correlates with inflammatory status [29]. According to these findings, intestinal flora imbalance may have a role in the occurrence and progression of RA, and a rise in pathogenic bacteria and a decrease in probiotic bacteria are crucial factors in the disease’s development.

Previous studies, both in plasma metabolic profiles and in fecal metabolic analysis, have shown that RA patients have considerable alterations in metabolites and metabolic pathways [11, 25, 30]. In our findings, disturbances in glycerophospholipid metabolism and phenylalanine metabolism pathways which are enriched by differential metabolites in RA patients, suggested that lipid metabolism and amino acid metabolism pathways apparently play a key role in the initiation and progression of RA. Consistent with our result, Yu et al. also found dysregulation of glycerophospholipid metabolism and amino acid metabolic pathways in RA patients [11]. More importantly, we also discovered a strong correlation between metabolites and clinical characteristics, with the metabolite glycerophosphocholine showing a significant negative correlation with CRP, while the metabolites L-Phenylalanine and trans-cinnamate showed a significant positive correlation with CRP. DAS28 and ESR showed significant positive correlations with metabolites L-Phenylalanine and alpha-N-Phenylacetyl-L-glutamine and negative correlations with glycerophosphocholine, which justified the close association of metabolites with CRP in previous studies, revealing that the activation of inflammatory factors during inflammatory states may influence the metabolic levels of metabolites [31]. In addition, we noted that the clinical data was associated with intestinal flora, with *Cystobasidium* and *DTU089* genera showed demonstrating a significant positive correlation with RF, and DAS28 being positively correlated with *Issatchenkia* genus and CRP expression levels and negatively correlated with *Sutterella*. Correlation analysis revealed that plasma metabolites are significantly dysregulated in RA patients and correlate and interact with intestinal flora and clinical features.

Inflammatory factors such as IL-7, IL-6 and tumor necrosis factor play an essential role in the activation of the inflammatory response in RA [32–34]. Our results showed that differentially expressed genes were enriched in the TGF-beta signaling pathway, the IL-17 signaling pathway, the MAPK signaling pathway, indicating that the inflammatory response pathway was disrupted in RA. A study reported that the MAPK signaling pathway was involved in cellular pathways in diseases such as RA, which was in accordance with our results [35]. Previous studies have described the involvement of interferons in a number of autoimmune diseases, including RA and SLE. Macías-Segura et al. showed that gene expression of type 1 interferon signaling was associated with autoantibody production in RA [36–38]. Consistent with the results of our GO enrichment analysis, significant dysregulation of type 1 interferon, platelet degradation, cell differentiation, and inflammatory response was demonstrated in RA patients. Importantly, we found that the results of KEGG enrichment analysis of differential genes are coincident with the results of KEGG analysis of differential metabolites, implying gene and metabolite interactions.

One of the highlights of our study was the multi-omics combination analysis of the potential associations among control, NORA, and CRA patients. Nevertheless, we noted the limitations of our study. First, the small sample size of our study may limit the reliability and accuracy of our findings, which will need to be investigated in multicenter cohort and experimental studies to further validate our findings. Second, although our study elucidated the progression of RA from molecular layer to metabolic level, we need to gain insight into the intrinsic associations between multiple omics to reveal the essential role of glycerophospholipid metabolism in the development of RA.

## Conclusion

In summary, comprehensive multi-omics analysis demonstrates that there is an inextricable link between control, NORA, and CRA, that intestinal flora interacts with plasma metabolites, and that the differential core gene may influence changes in glycerophospholipid metabolism pathway. These findings suggest that glycerophospholipid metabolism is involved in the pathogenesis and development of RA from the molecular to the metabolite level and plays a significant role in RA pathogenesis, offering suggestions for early clinical diagnosis and therapeutic approaches.

### Supplementary Information

**Additional file 1: Supplementary Material 1.**

**Additional file 2: Supplementary Material 2.**

**Additional file 3: Supplementary Figure 1.** Analysis of plasma metabolic profiles between control, NORA and CRA patients. (A, B) OPLA-DA analysis showed the 111 differential metabolites between NORA and control in anionic and cationic mode, respectively. (C) Volcano plot demonstrated 70 upregulated and 41 downregulated of 111 differential metabolites. (D, E) OPLA-DA analysis exhibited 110 differentially significant metabolites between CRA and control in anionic and cationic modes, separately. (F) Volcano plot showed 41 upregulated and 69 downregulated of 110 differential metabolites. (G) Volcano plot displayed 10 upregulated and 19 downregulated metabolites out of 29 differential metabolites. (H-J) Heatmaps demonstrated the clustering of 111, 110, and 29 differential metabolites, respectively, showing a clear distinction between these differential metabolites in groups. (K) Bubble plot demonstrated 111 differential metabolites enriched in 5 metabolic pathways between NORA and control. (L) Bubble plot showed 110 differential metabolites significantly enriched in 9 pathways between CRA and control. (M, N) Scatter plots demonstrated relative abundance of alpha-N-Phenylacetyl-L-glutamine and L-Phenylalanine on phenylalanine metabolism pathway between control, NORA and CRA. (O) Correlation heatmap showed the interrelationship between the 6 differential metabolites.

**Additional file 4: Supplementary Figure 2.** Gut bacterial community composition among control, NORA and CRA groups. (A) Venn diagram showed the common 133 species between the three groups. (B-D) Bar graphs showed the changes in abundance of *P_Firmicutes*, *P_Bacteroidota*, and *P_Actinobacteriota* among the three groups, respectively, although they did not differ from each other. (E) Stacked bar graph exhibited the community composition at the family level between three groups. (F-J) Bar graphs showed the relative abundance of *f_Pasteurellaceae*, *f_Acidaminococcaceae*, *f_Bacteroidaceae*, *f_Prevotellaceae* and *f_Lactobacillaceae*, respectively.

**Additional file 5: Supplementary Figure 3.** Gut bacterial community diversity among control, NORA and CRA groups. (A-F) The bar graphs showed the relative abundance of ace, chao, sobs, shannon, simpson and pd indexes between the three groups, although there was no difference in community richness and diversity among the three groups. (G, H) PCoA in beta diversity analysis demonstrated the community structure between CRA and control, and between NORA and CRA, respectively, indicating similar community between them. Adonis between-group difference test using bray-curtis distance algorithm, analyzed by number of 999 substitutions. (I-K) Linear discriminant analysis (LDA) demonstrated the importance of species from phylum to genus level among control, NORA, and CRA groups.

**Additional file 6: Supplementary Figure 4.** Intestinal fungal community composition and diversity between controls, NORA and CRA groups. (A) Venn diagram showed the common 64 species among three groups. (B) Stacked bar showed the community composition at the family level among control, NORA and CRA. (C-F) The bar graphs exhibited the abundance of *f_Cladosporiaceae*, *f_Phaffomycetaceae*, *f_Debaryomycetaceae*, and *f_Didymellaceae* among the three groups, respectively, indicating the differences in abundance among the three groups. (G, H) PCoA demonstrated similar community structure between NORA and control, and between NORA and CRA, respectively. Adonis between-group difference test using bray-curtis distance algorithm, analyzed by number of 999 substitutions.

**Additional file 7: Supplementary Figure 5.** Gene enrichment analysis between controls, NORA and CRA groups. GSEA enrichment analysis showed 8 gene sets that were significantly enriched in control compared to NORA, including (A)glycosylphosphatidylinositol gpi anchor biosynthesis, (B)calcium signaling pathway, (C)taste transduction, (D)neuroactive ligand receptor interaction, (E)RNA polymerase, (F)arachidonic acid metabolism, (G)hedgehog signaling pathway, and (H)basal cell carcinoma. (I, J) The bubble plots displayed KEGG and GO enrichment analysis for 196 differentially expressed genes between NORA and control, respectively. (K, L) The bubble plots displayed KEGG and GO enrichment analysis for 211 differentially expressed genes between CRA and control, respectively.

**Additional file 8: Supplementary Figure 6.** (A) Correlation heat map showed the interactions and associations between differential flora, differential metabolites and clinical features.

**Additional file 9: Supplementary Figure 7. **Signatures were selected based on LASSO machine algorithms and random forest. (A) ROC analysis of 7 species of differential bacteria. (B) ROC analysis of 8 differential fungi. (C) ROC analysis of 6 differential metabolites. (D) Graph of features screened based on the LASSO machine algorithm. (E) Random forest model showed the evaluation of the top important features (*n*=6, AUC=0.856). (F) 6 crucial signatures were selected based on applying random forest algorithm.

**Additional file 10: Supplementary Table 1. **Clinical characteristics of the subjects included in our study.** Supplementary Table 2. **Enrichment pathway of 6 differentially significant metabolites between NORA and CRA patients.** Supplementary Table 3.** 9 gene sets were significantly enriched in the control, based on all protein-coding genes by GSEA analysis between NORA and control.** Supplementary Table 4. **Significant enrichment of 3 gene sets in control, 16 gene sets in CRA, based on all protein-coding genes between CRA and controls by GSEA analysis.** Supplementary Table 5. **KEGG and GO enrichment analysis of 196 differentially expressed genes between NORA and control.** Supplementary Table 6. **KEGG and GO enrichment analysis of 211 differentially expressed genes between CRA and control.** Supplementary Table 7. **KEGG and GO enrichment analysis of 40 differentially expressed genes between NORA and CRA patients.

## Data Availability

Our raw data have been uploaded to the database (NGDC database and PRJCA011639) and will be made public as soon as possible and, if necessary, will be sent to the relevant applicants after a reasonable request to the corresponding author.

## Abbreviations

RA: Rheumatoid arthritis
NORA: New-onset rheumatoid arthritis
CRA: Chronic rheumatoid arthritis
ITS: Internally transcribed spacer
LASSO: Least Absolute Shrinkage and Selection Operator
ACHE: Acetylcholinesterase
PBMCs: Peripheral blood mononuclear cells
NSAIDs: Non-steroidal anti-inflammatory drugs
ACR: American College of Rheumatology
EULAR: European League of Rheumatology
RF: Rheumatoid factor
ESR: Erythrocyte sedimentation rate
CRP: C-reactive protein
TJC28: 28 Tender joints count
SJC28: 28 Swollen joints count
DAS28: Disease activity score of 28 joints
IL-6: Interleukin-6
ASVs: Amplicon sequence variants
LC-MS: Liquid chromatography-tandem mass spectrometry
RSD: Relative standard deviations
LDA: Linear discriminant analysis
LEfSe: Linear discriminant analysis effect size
OPLS-DA: Orthogonal partial least squares discriminant analysis
KEGG: Kyoto Encyclopedia of Genes and Genomes
GO: Gene Ontology
S-lysoPC: 1-Stearoyl-2-hydroxy-sn-glycero-3- phosphocholine
PCoA: Principal co-ordinates analysis
GSEA: Gene Set Enrichment Analysis
FN1: Fibronectin 1
AQP1: Aquaporin 1
LYPLA1: Lysophospholipase 1
GCKR: Glucokinase regulator
TAT: Tyrosine aminotransferase
SLE: Systemic lupus erythematosus
