# Supplementary material for: Comprehensive multi-omics analysis reveals the core role of glycerophospholipid metabolism in rheumatoid arthritis development
Source: Arthritis Res Ther. 2023 Dec 15;25:246. doi: 10.1186/s13075-023-03208-2 (PMC10722724; doi:10.1186/s13075-023-03208-2)
Supplement: Supplementary file 1 — Additional file 1: Supplementary Material 1. [file 13075_2023_3208_MOESM1_ESM.pdf]

Supplementary Material 1

Stool sample collection

Stool samples were collected from patients with RA on admission to the rheumatology department and were split and processed into 1.5 ml EP tube within two hours, subsequently frozen in liquid nitrogen for 5 minutes before being removed and stored at -80°C refrigerator until use.

DNA extraction, PCR amplification and Illumina sequencing

Total DNA extraction from microbial communities was performed according to the instructions of the E.Z.N.A.® soil DNA kit (Omega Bio-tek, Norcross, GA, U.S.), and the quality of DNA extraction was determined using 1% agarose gel electrophoresis, and DNA concentration and purity were determined using NanoDrop2000. 16S rRNA sequencing V3-V4 variable region PCR amplification primers were 338F (5'-ACTCCTACGGGAGGCAGCAG-3') and 806R (5'-GGACTACHVGGGTWTCTAAT-3') and ITS amplification primers were ITS1F (5'-CTTGGTCATTTAGAGGAAGTAA-3') and ITS2R (5'-GCTGCGTTCATCGATGC-3'). PCR products were recovered using 2% agarose gels, purified using AxyPrep DNA Gel Extraction Kit (Axygen Biosciences, Union City, CA, USA), and sequenced using Illumina Miseq PE300 platform (Majorbio Bio-Pharm Technology Co. Ltd. (Shanghai, China).).

Data processing

The raw data obtained by up-sequencing were quality-controlled and spliced by fastp (https://github.com/OpenGene/fastp, version 0.20.0) and FLASH (http://www.cbcb.umd.edu/software/flash，version 1.2.7) software, respectively, and subsequently the optimized sequences after quality-controlled splicing were noise-reduced using the DADA2 plug-in in the Qiime2 process to obtain amplicon sequence variants (ASVs). The ASVs were analysed taxonomically for species through the Sliva 16S rRNA database (v 138) using the Naive baye classifier in Qiime2. Prior to data analysis, we sampled each sample according to the minimum number of sequences, and subsequently the number of valid sequences obtained was used for the intestinal flora diversity and species composition analysis.
