# Supplementary material for: Comprehensive multi-omics analysis reveals the core role of glycerophospholipid metabolism in rheumatoid arthritis development
Source: Arthritis Res Ther. 2023 Dec 15;25:246. doi: 10.1186/s13075-023-03208-2 (PMC10722724; doi:10.1186/s13075-023-03208-2)
Supplement: Supplementary file 2 — Additional file 2: Supplementary Material 2. [file 13075_2023_3208_MOESM2_ESM.pdf]

Supplementary Material 2

Blood and stool sample collection

Morning fasting blood samples were collected from each participant and centrifuged at 3500 rpm for 10 minutes, followed by transferring the supernatant to a 1.5 ml EP tube and centrifuging at high speed for 12,000 rpm for 10 minutes. Finally, aspirated the supernatant into another EP tube and stored at -80°C in the refrigerator until use.

Non-targeted LC-MS/MS analysis

100 μL of liquid sample was added to a 1.5 mL centrifuge tube, followed by 400 μL of extraction solution (acetonitrile: methanol = 1:1), mixed for 30 s and then extracted by low-temperature sonication for 30 min (5°C, 40 KHz), the sample was left at -20°C for 30 min, centrifuged at 4°C, 13000 g for 15 min, the supernatant was removed, blown dry under nitrogen, 120 μL of re-solution (acetonitrile: water = 1:1), low-temperature ultrasonic extraction for 5 min (5°C, 40 KHz), centrifugation at 13,000 g for 5 min at 4°C, and transferring the supernatant to the injection vial with internal cannula for analysis. In the on-line analysis, 1 QC sample was inserted in every 10 samples to observe the reproducibility throughout the analysis process. The analytical instrument was an ultra-performance liquid chromatography tandem time of flight mass spectrometry UPLC -TripleTOF system from AB SCIEX.

Data processing and analysis

The raw data from the assays were pre-processed by the metabolomics software Progenesis QI (Waters Corporation，Milford，USA) to obtain a data matrix, which retained at least 80% of the non-zero values in at least one set of samples and filled in the gaps with the minimum values, while removing variables with a relative standard deviation (RSD) >30% for QC samples to obtain the final data matrix for subsequent analysis. The mass spectrometry information was also matched with the metabolic public databases HMDB (http: //www.hmdb.ca/) and Metlin (https: //metlin.scripps.edu/) databases to obtain metabolite information for subsequent differential metabolite analysis and pathway enrichment analysis. The variable weight values (VIP) and Wilcox test p-values obtained from the OPLS-DA model were used to determine that metabolites with VIP>1 and p<0.05 were significantly different metabolites. The pathways involved in differential metabolites were also obtained using metabolic pathway annotation from the KEGG database (https://www.kegg.jp/kegg/pathway.html). The Python package scipy.stats was used for pathway enrichment analysis and the relevant biological pathways were obtained by Fisher's exact test.
