# Supplementary figures and images for: Comprehensive multi-omics analysis reveals the core role of glycerophospholipid metabolism in rheumatoid arthritis development
Source: Arthritis Res Ther. 2023 Dec 15;25:246. doi: 10.1186/s13075-023-03208-2 (PMC10722724; doi:10.1186/s13075-023-03208-2)

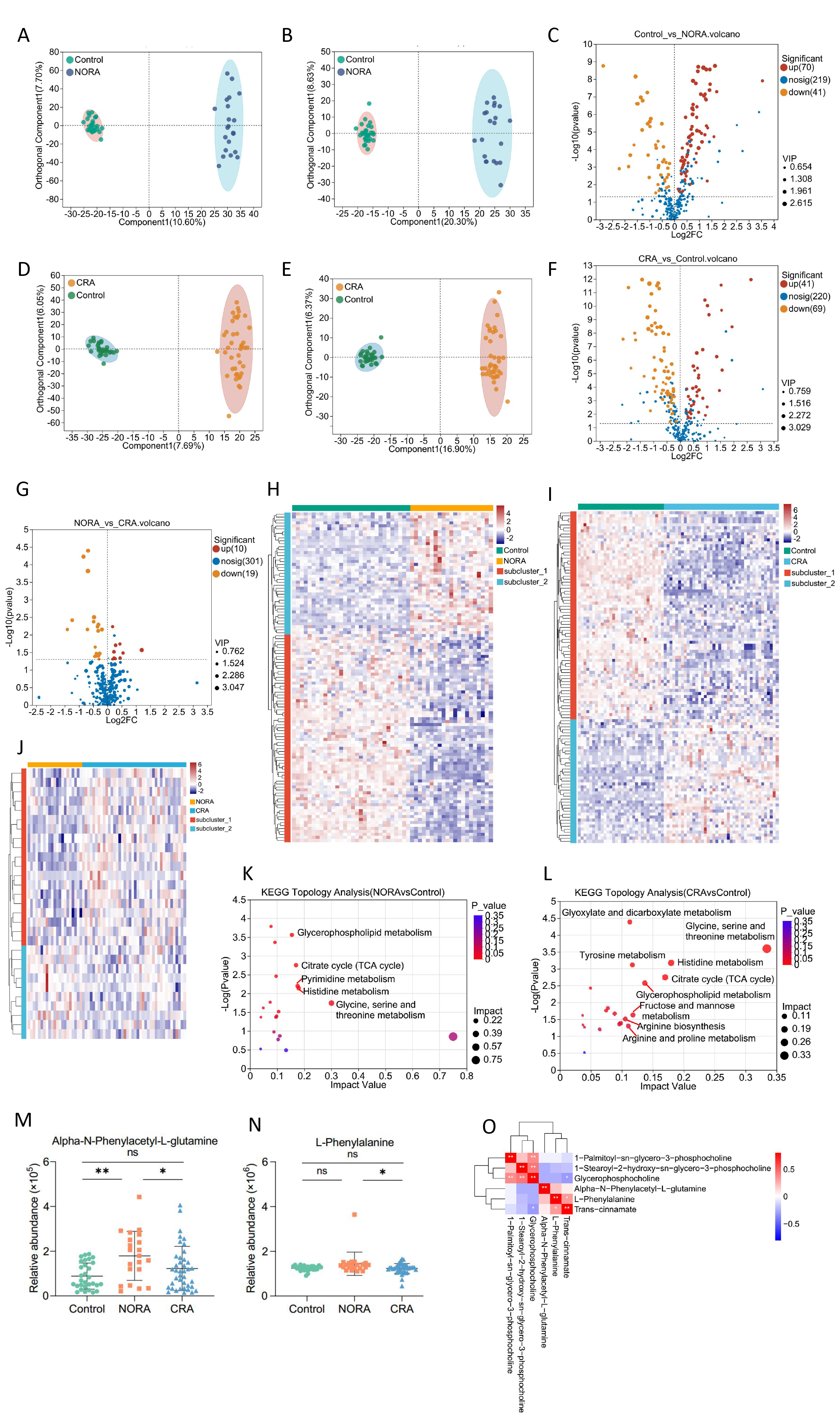

Supplement: Supplementary file 3 — Additional file 3: Supplementary Figure 1. Analysis of plasma metabolic profiles between control, NORA and CRA patients. (A, B) OPLA-DA analysis showed the 111 differential metabolites between NORA and control in anionic and cationic mode, respectively. (C) Volcano plot demonstrated 70 upregulated and 41 downregulated of 111 differential metabolites. (D, E) OPLA-DA analysis exhibited 110 differentially significant metabolites between CRA and control in anionic and cationic modes, separately. (F) Volcano plot showed 41 upregulated and 69 downregulated of 110 differential metabolites. (G) Volcano plot displayed 10 upregulated and 19 downregulated metabolites out of 29 differential metabolites. (H-J) Heatmaps demonstrated the clustering of 111, 110, and 29 differential metabolites, respectively, showing a clear distinction between these differential metabolites in groups. (K) Bubble plot demonstrated 111 differential metabolites enriched in 5 metabolic pathways between NORA and control. (L) Bubble plot showed 110 differential metabolites significantly enriched in 9 pathways between CRA and control. (M, N) Scatter plots demonstrated relative abundance of alpha-N-Phenylacetyl-L-glutamine and L-Phenylalanine on phenylalanine metabolism pathway between control, NORA and CRA. (O) Correlation heatmap showed the interrelationship between the 6 differential metabolites. [file 13075_2023_3208_MOESM3_ESM.tif]

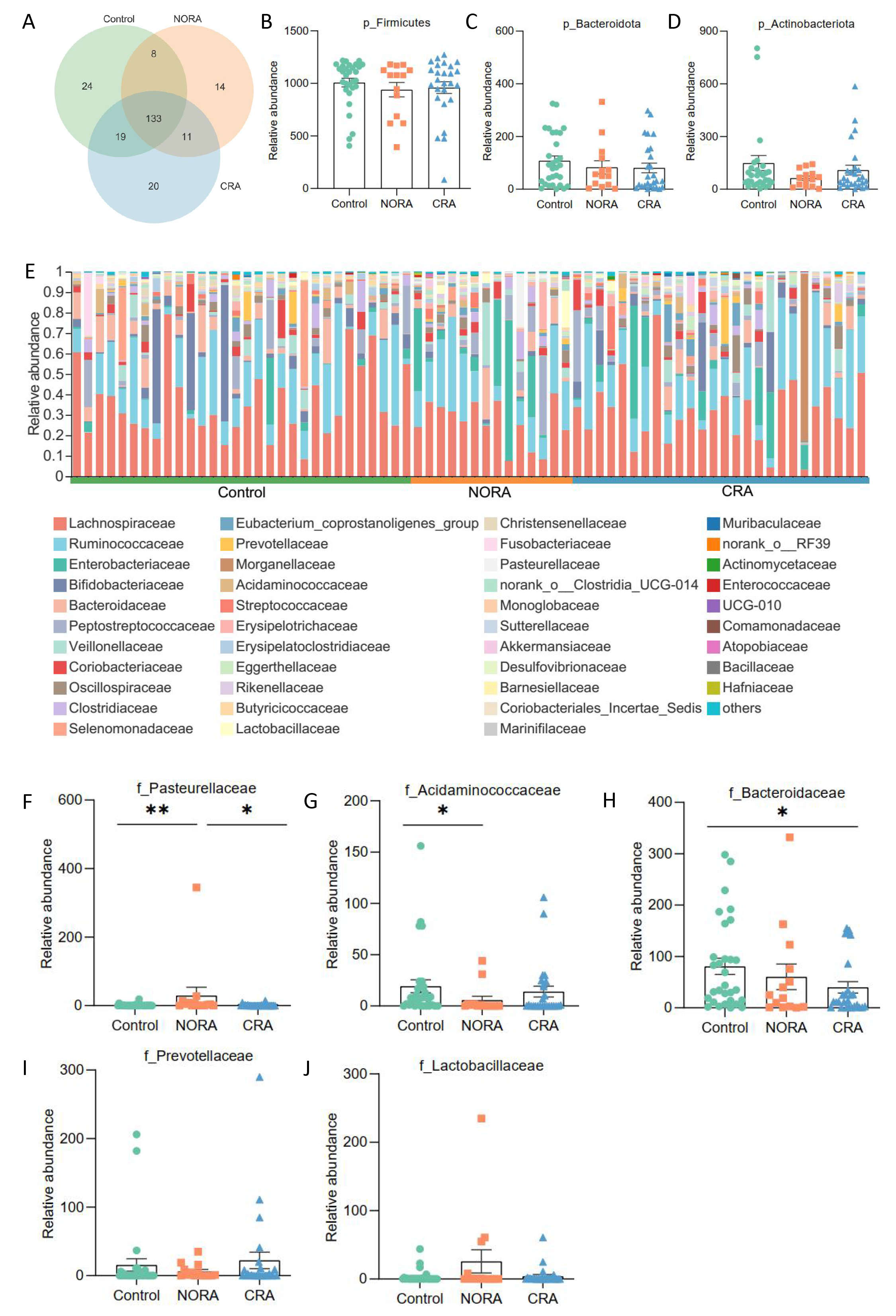

Supplement: Supplementary file 4 — Additional file 4: Supplementary Figure 2. Gut bacterial community composition among control, NORA and CRA groups. (A) Venn diagram showed the common 133 species between the three groups. (B-D) Bar graphs showed the changes in abundance of P_Firmicutes, P_Bacteroidota, and P_Actinobacteriota among the three groups, respectively, although they did not differ from each other. (E) Stacked bar graph exhibited the community composition at the family level between three groups. (F-J) Bar graphs showed the relative abundance of f_Pasteurellaceae, f_Acidaminococcaceae, f_Bacteroidaceae, f_Prevotellaceae and f_Lactobacillaceae, respectively. [file 13075_2023_3208_MOESM4_ESM.tif]

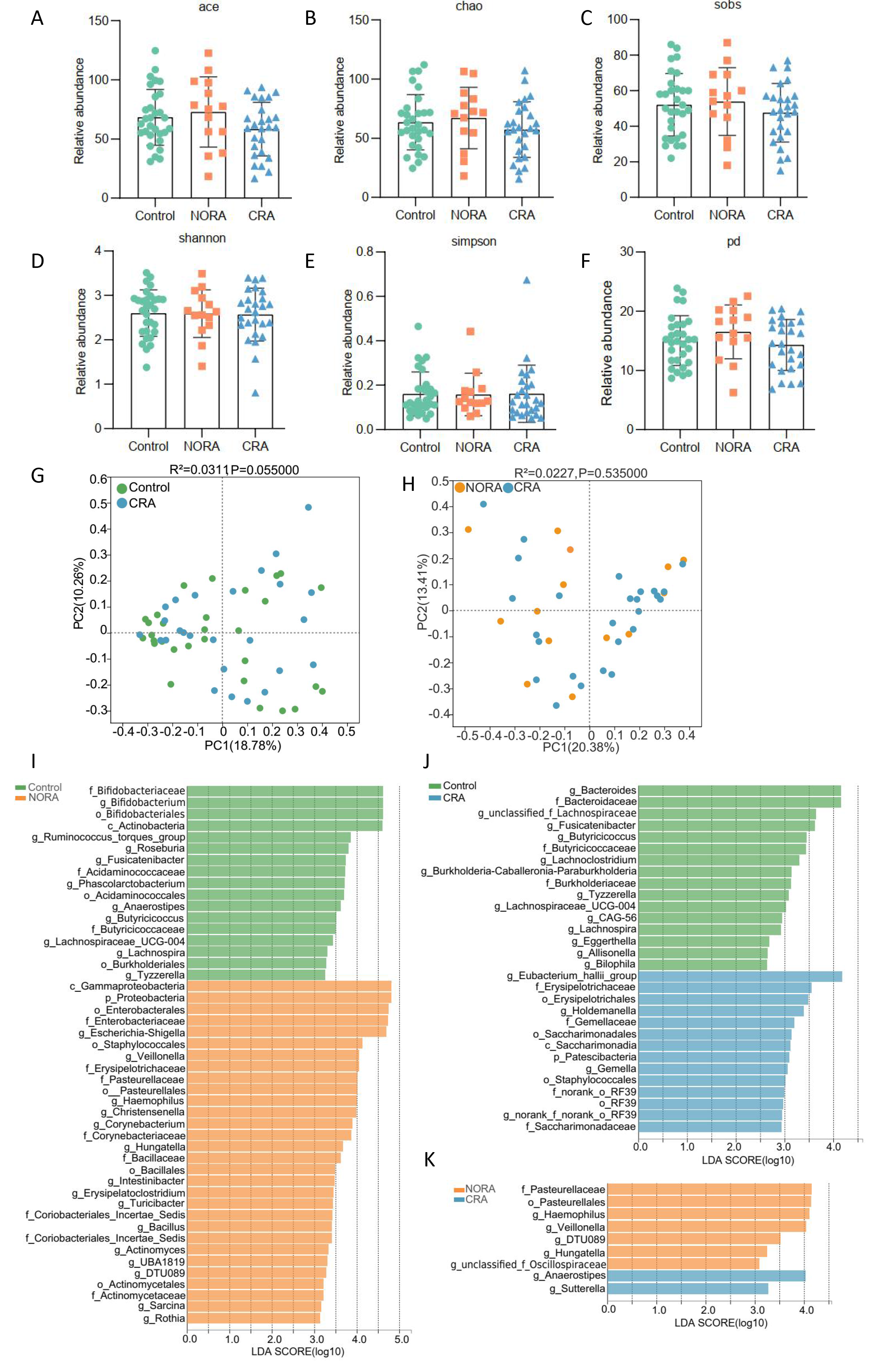

Supplement: Supplementary file 5 — Additional file 5: Supplementary Figure 3. Gut bacterial community diversity among control, NORA and CRA groups. (A-F) The bar graphs showed the relative abundance of ace, chao, sobs, shannon, simpson and pd indexes between the three groups, although there was no difference in community richness and diversity among the three groups. (G, H) PCoA in beta diversity analysis demonstrated the community structure between CRA and control, and between NORA and CRA, respectively, indicating similar community between them. Adonis between-group difference test using bray-curtis distance algorithm, analyzed by number of 999 substitutions. (I-K) Linear discriminant analysis (LDA) demonstrated the importance of species from phylum to genus level among control, NORA, and CRA groups. [file 13075_2023_3208_MOESM5_ESM.tif]

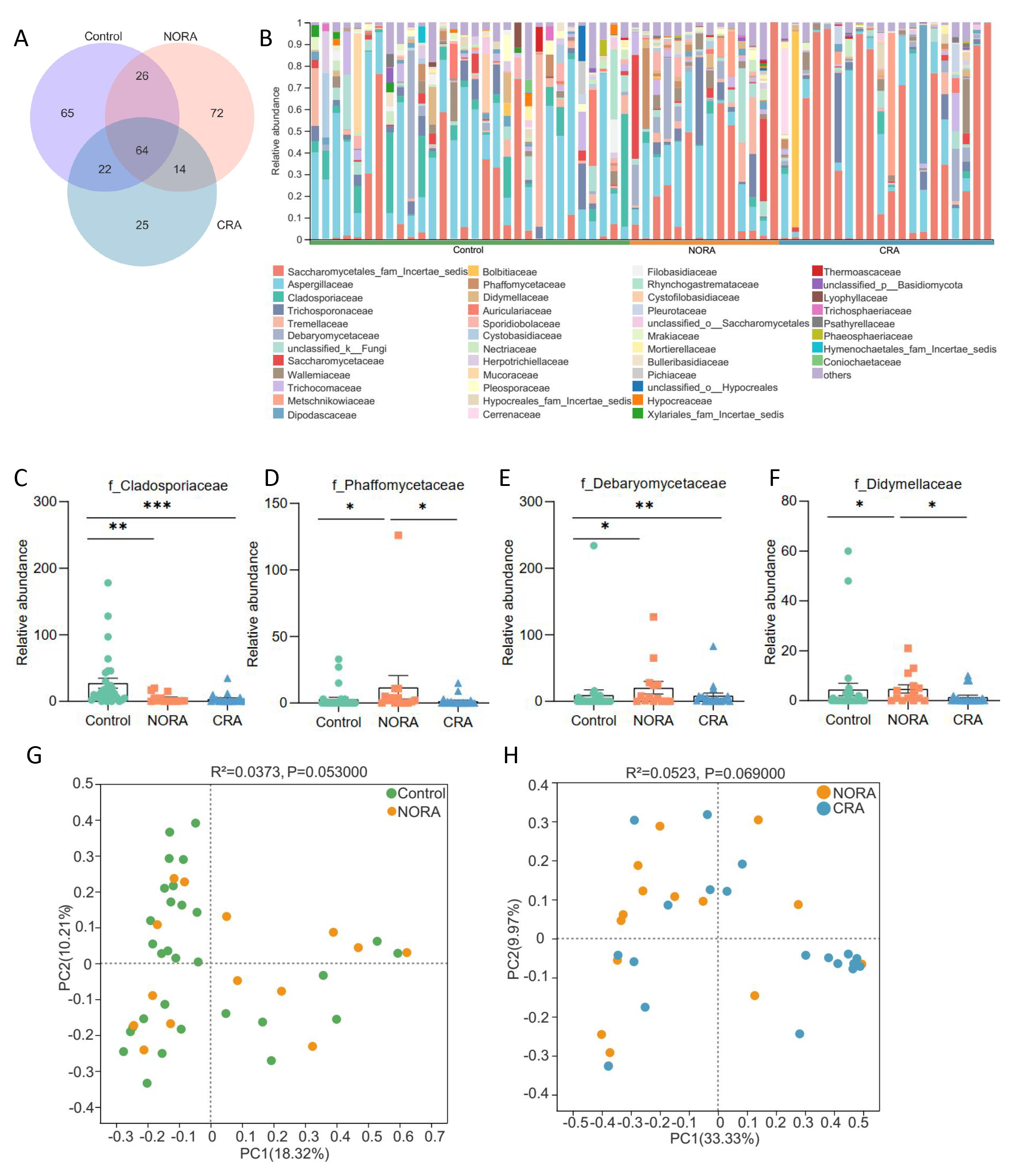

Supplement: Supplementary file 6 — Additional file 6: Supplementary Figure 4. Intestinal fungal community composition and diversity between controls, NORA and CRA groups. (A) Venn diagram showed the common 64 species among three groups. (B) Stacked bar showed the community composition at the family level among control, NORA and CRA. (C-F) The bar graphs exhibited the abundance of f_Cladosporiaceae, f_Phaffomycetaceae, f_Debaryomycetaceae, and f_Didymellaceae among the three groups, respectively, indicating the differences in abundance among the three groups. (G, H) PCoA demonstrated similar community structure between NORA and control, and between NORA and CRA, respectively. Adonis between-group difference test using bray-curtis distance algorithm, analyzed by number of 999 substitutions. [file 13075_2023_3208_MOESM6_ESM.tif]

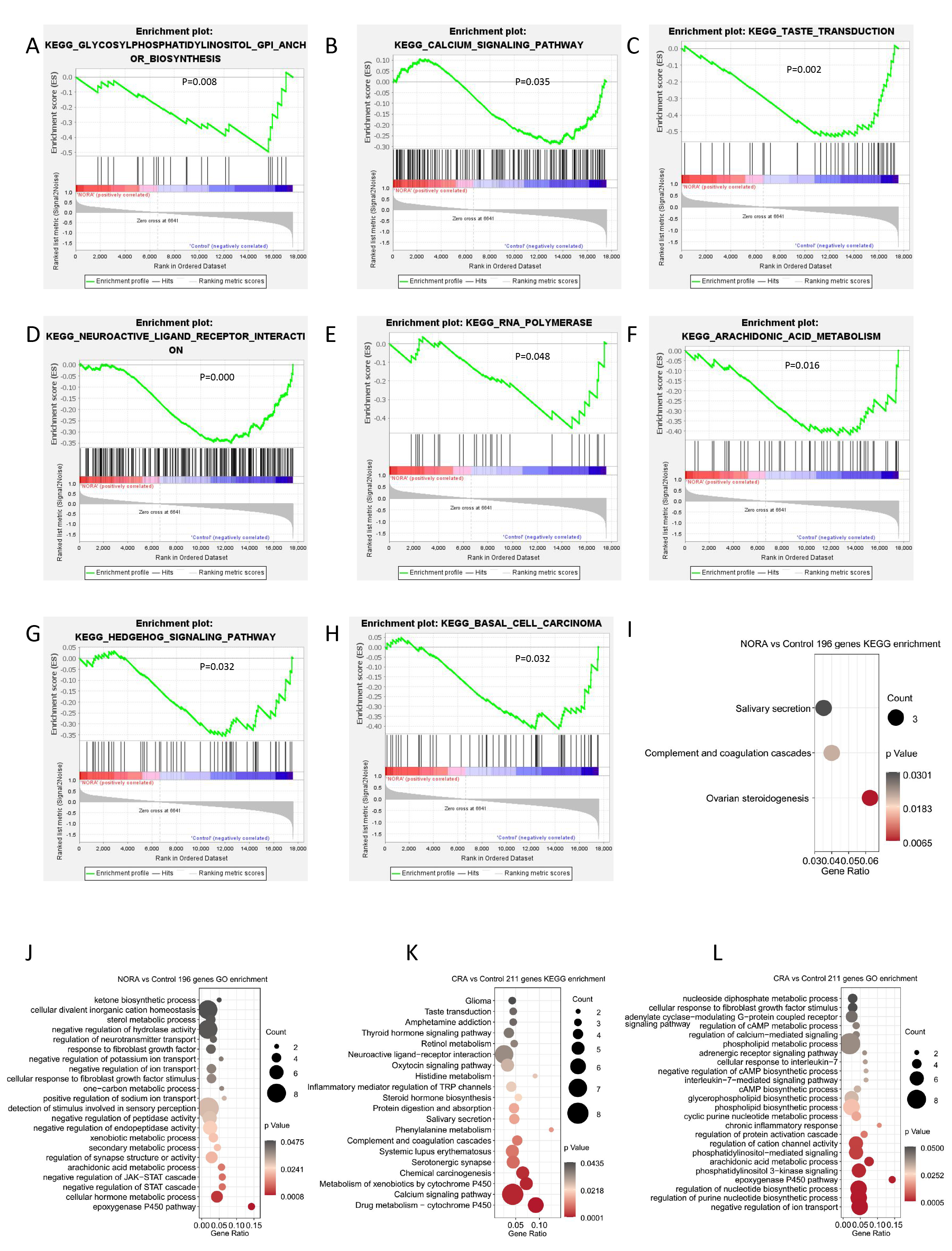

Supplement: Supplementary file 7 — Additional file 7: Supplementary Figure 5. Gene enrichment analysis between controls, NORA and CRA groups. GSEA enrichment analysis showed 8 gene sets that were significantly enriched in control compared to NORA, including (A)glycosylphosphatidylinositol gpi anchor biosynthesis, (B)calcium signaling pathway, (C)taste transduction, (D)neuroactive ligand receptor interaction, (E)RNA polymerase, (F)arachidonic acid metabolism, (G)hedgehog signaling pathway, and (H)basal cell carcinoma. (I, J) The bubble plots displayed KEGG and GO enrichment analysis for 196 differentially expressed genes between NORA and control, respectively. (K, L) The bubble plots displayed KEGG and GO enrichment analysis for 211 differentially expressed genes between CRA and control, respectively. [file 13075_2023_3208_MOESM7_ESM.tif]

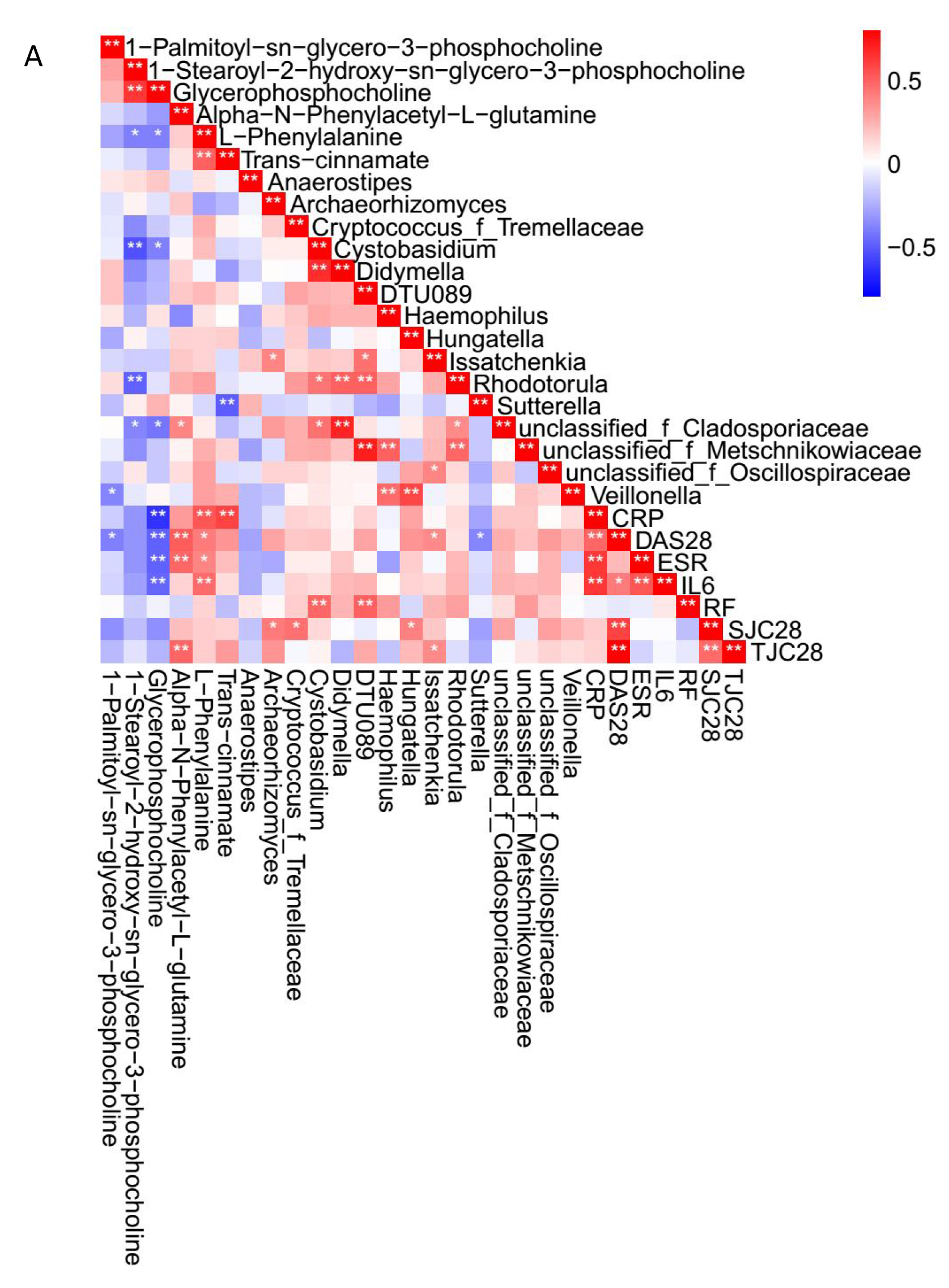

Supplement: Supplementary file 8 — Additional file 8: Supplementary Figure 6. (A) Correlation heat map showed the interactions and associations between differential flora, differential metabolites and clinical features. [file 13075_2023_3208_MOESM8_ESM.tif]

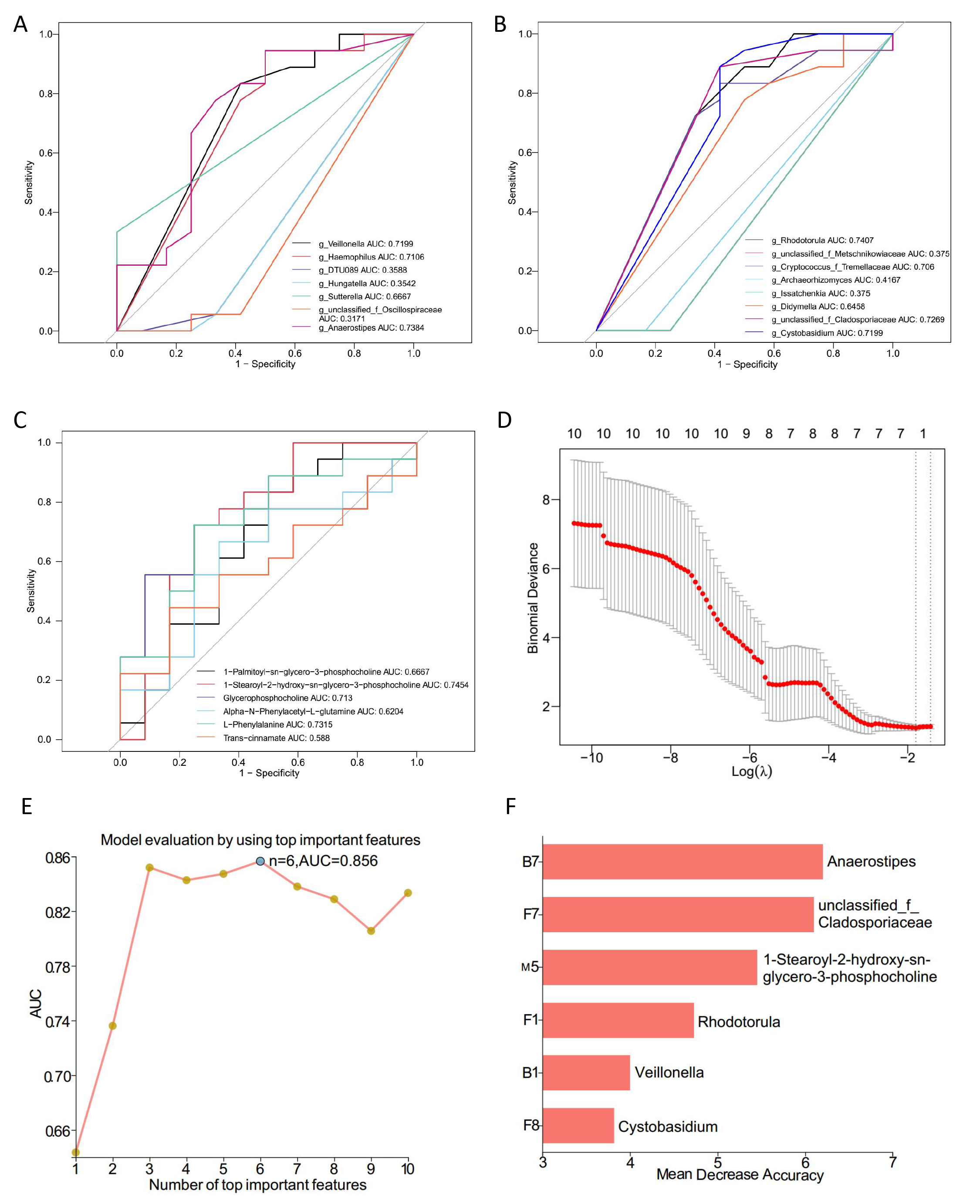

Supplement: Supplementary file 9 — Additional file 9: Supplementary Figure 7. Signatures were selected based on LASSO machine algorithms and random forest. (A) ROC analysis of 7 species of differential bacteria. (B) ROC analysis of 8 differential fungi. (C) ROC analysis of 6 differential metabolites. (D) Graph of features screened based on the LASSO machine algorithm. (E) Random forest model showed the evaluation of the top important features (n=6, AUC=0.856). (F) 6 crucial signatures were selected based on applying random forest algorithm. [file 13075_2023_3208_MOESM9_ESM.tif]
