# Supplementary material for: Comprehensive multi-omics analysis reveals the core role of glycerophospholipid metabolism in rheumatoid arthritis development
Source: Arthritis Res Ther. 2023 Dec 15;25:246. doi: 10.1186/s13075-023-03208-2 (PMC10722724; doi:10.1186/s13075-023-03208-2)
Supplement: Supplementary file 10 — Additional file 10: Supplementary Table 1. Clinical characteristics of the subjects included in our study. Supplementary Table 2. Enrichment pathway of 6 differentially significant metabolites between NORA and CRA patients. Supplementary Table 3. 9 gene sets were significantly enriched in the control, based on all protein-coding genes by GSEA analysis between NORA and control. Supplementary Table 4. Significant enrichment of 3 gene sets in control, 16 gene sets in CRA, based on all protein-coding genes between CRA and controls by GSEA analysis. Supplementary Table 5. KEGG and GO enrichment analysis of 196 differentially expressed genes between NORA and control. Supplementary Table 6. KEGG and GO enrichment analysis of 211 differentially expressed genes between CRA and control. Supplementary Table 7. KEGG and GO enrichment analysis of 40 differentially expressed genes between NORA and CRA patients. [file 13075_2023_3208_MOESM10_ESM.pdf]

Supplementary Table 1. Clinical characteristics of the subjects were included in our study.

Characteristics	Discovery cohort		P	Control (n=75)	Validation cohort		P
	NORA (n=34)	CRA (n=53)			NORA (n=8)	CRA (n=18)	
Age (year)	56.74±12.98	57.94±10.55	0.635	51.61±7.72	61.25±8.48	58.28±8.27	0.410
Female,sex (n)	20	41	0.065	31	5	10	1.000
CRP (mg/L)	37.12±39.16	36.49±32.85	0.548	——	47.91±55.31	59.98±48.72	0.291
ESR (mm/h)	56.73±35.08	63.08±30.02	0.376	——	67.63±37.36	74.39±31.73	0.639
RF (U/ml)	269.51±217.67	277.74±403.03	0.401	——	231.81±453.51	359.60±230.48	0.046*
SJC28	10.10±7.41	6.33±6.92	0.014*	——	15.50±9.70	8.61±10.04	0.083
TJC28	11.65±7.75	9.72±8.19	0.218	——	16.00±11.05	10.39±10.99	0.261
DAS28(3)	5.56±1.39	5.35±1.23	0.358	——	5.79±1.69	4.99±2.10	0.350
IL-6 (pg/mL)	84.42±80.31	65.42±125.90	0.032*	——	——	——	——

Date presented as number mean±standard deviation.

NORA, new-onset rheumatoid arthritis; CRA, chronic rheumatoid arthritis; CRP, C-reactive protein; ESR, erythrocyte sedimentation rate; RF, rheumatoid factors; SJC28, 28 swollen joints count; TJC28, 28 tender joints count; DAS28, disease activity score; DAS28(3) = [0.56*sqrt(t28) + 0.28*sqrt(sw28) + 0.70*Ln(ESR)]*1.08 + 0.16; IL-6, Interleukin 6; *, $P \leq 0.05$.

Supplementary Table 2. Enrichment pathway of 6 differentially significant metabolites between NORA and CRA patients.

Metabolite	VIP	FC	P value
L-Phenylalanine	2.0842	1.1692	0.0305*
Trans-cinnamate	1.5284	1.3520	0.0183*
Alpha-N-Phenylacetyl-L-glutamine	1.6636	1.4605	0.0330*
1-Palmitoyl-sn-glycero-3-phosphocholine	1.8082	0.6142	0.0071**
1-Stearoyl-2-hydroxy-sn-glycero-3-phosphocholine	3.0471	0.6216	0.0002***
Glycerophosphocholine	1.9527	0.714	0.0042**

NORA, new-onset rheumatoid arthritis; CRA, chronic rheumatoid arthritis. *, $P < 0.05$; **, $P \leq 0.01$, ***, $P \leq 0.001$.

Supplementary Table 3. 9 gene sets were significantly enriched in the control, based on all protein-coding genes by GSEA analysis between NORA and control.

NAME	SIZE	ES	NES	P value
KEGG_NEUROACTIVE_LIGAND_RECEPTOR_INTERACTION	216	-0.35	-1.59	0.000
KEGG_TASTE_TRANSDUCTION	42	-0.53	-1.83	0.002
KEGG_GLYCOSYLPHOSPHATIDYLINOSITOL_ GPI_ANCHOR_BIOSYNTHESIS	24	-0.50	-1.66	0.008
KEGG_ARACHIDONIC_ACID_METABOLISM	48	-0.42	-1.58	0.016
KEGG_GLYCEROPHOSPHOLIPID_METABOLISM	73	-0.37	-1.54	0.020
KEGG_HEDGEHOG_SIGNALING_PATHWAY	53	-0.36	-1.43	0.032
KEGG_BASAL_CELL_CARCINOMA	54	-0.41	-1.54	0.032
KEGG_CALCIUM_SIGNALING_PATHWAY	162	-0.29	-1.36	0.035
KEGG_RNA_POLYMERASE	28	-0.45	-1.47	0.048

NORA, new-onset rheumatoid arthritis; CRA, chronic rheumatoid arthritis; GSEA, Gene Set Enrichment Analysis.

Supplementary Table 4. Significant enrichment of 3 gene sets in control, 16 gene sets in CRA, based on all protein-coding genes between CRA and controls by GSEA analysis.

3 gene sets enriched in control				
NAME	SIZE	ES	NES	P-value
KEGG_TASTE_TRANSDUCTION	42	-0.57	-1.74	0.012
KEGG_LINOLEIC_ACID_METABOLISM	25	-0.57	-1.62	0.002
KEGG_ARRHYTHMOGENIC_RIGHT_VENTRICULAR_CARDIOMYOPATHY_ARVC	73	-0.33	-1.40	0.038
16 gene sets enriched in CRA				
NAME	SIZE	ES	NES	P value
KEGG_CYSTEINE_AND_METHIONINE_METABOLISM	33	0.52	1.83	0.002
KEGG_PARKINSONS_DISEASE	121	0.62	1.81	0.034
KEGG_OXIDATIVE_PHOSPHORYLATION	126	0.61	1.80	0.036
KEGG_HUNTINGTONS_DISEASE	173	0.49	1.75	0.038
KEGG_LYSOSOME	118	0.52	1.74	0.006
KEGG_ALZHEIMERS_DISEASE	158	0.48	1.73	0.045
KEGG_GLUTATHIONE_METABOLISM	43	0.52	1.73	0.002
KEGG_CITRATE_CYCLE_TCA_CYCLE	28	0.62	1.73	0.016
KEGG_PROTEASOME	44	0.52	1.71	0.028
KEGG_ANTIGEN_PROCESSING_AND_PRESENTATION	69	0.48	1.67	0.010
KEGG_PROTEIN_EXPORT	23	0.59	1.67	0.029
KEGG_PATHOGENIC_ESCHERICHIA_COLI_INFECTION	54	0.52	1.65	0.021
KEGG_OOCYTE_MEIOSIS	107	0.38	1.59	0.014
KEGG_BIOSYNTHESIS_OF_UNSATURATED_FATTY_ACIDS	22	0.52	1.58	0.022
KEGG_PENTOSE_PHOSPHATE_PATHWAY	25	0.52	1.56	0.042
KEGG_SNARE_INTERACTIONS_IN_VESICULAR_TRANSPORT	38	0.42	1.52	0.047

Supplementary Table 5. KEGG and GO enrichment analysis of 196 differentially expressed genes between NORA and control.

ID	Pathway_Name	Count	Enrichment	p_Value
hsa04913	Ovarian steroidogenesis	3	0.0625	0.006492759
hsa04610	Complement and coagulation cascades	3	0.04	0.021789907
hsa04970	Salivary secretion	3	0.035294118	0.030145967
GO:0019373	epoxygenase P450 pathway	3	0.142857143	0.000771184
GO:0034754	cellular hormone metabolic process	5	0.043478261	0.003362224
GO:0046426	negative regulation of JAK-STAT cascade	3	0.058823529	0.009980307
GO:1904893	negative regulation of STAT cascade	3	0.058823529	0.009980307
GO:0019369	arachidonic acid metabolic process	3	0.056603774	0.011086064
GO:0050803	regulation of synapse structure or activity	5	0.028409091	0.01913541
GO:0019748	secondary metabolic process	3	0.044776119	0.020760376
GO:0006805	xenobiotic metabolic process	4	0.033333333	0.020971551
GO:0010951	negative regulation of endopeptidase activity	6	0.024	0.022504345
GO:0010466	negative regulation of peptidase activity	6	0.022813688	0.027947001
GO:0050906	detection of stimulus involved in sensory perception	9	0.018255578	0.029030265
GO:0010765	positive regulation of sodium ion transport	2	0.064516129	0.029796211
GO:0006730	one-carbon metabolic process	2	0.057142857	0.03727676
GO:0044344	cellular response to fibroblast growth factor stimulus	4	0.027777778	0.037446944
GO:0043271	negative regulation of ion transport	4	0.027586207	0.038257559
GO:0043267	negative regulation of potassium ion transport	2	0.055555556	0.039248174
GO:0071774	response to fibroblast growth factor	4	0.026490066	0.043332106
GO:0051588	regulation of neurotransmitter transport	3	0.033333333	0.04410979
GO:0051346	negative regulation of hydrolase activity	8	0.017621145	0.045733779
GO:0016125	sterol metabolic process	4	0.025974026	0.046004936
GO:0072503	cellular divalent inorganic cation homeostasis	8	0.01754386	0.046713745
GO:0042181	ketone biosynthetic process	2	0.05	0.047514063

Supplementary Table 6. KEGG and GO enrichment analysis of 211 differentially expressed genes between CRA and control.

ID	Pathway_Name	Count	Enrichment	p_Value
hsa00982	Drug metabolism - cytochrome P450	6	0.092307692	8.04E-05
hsa04020	Calcium signaling pathway	8	0.043715847	0.000966098
hsa00980	Metabolism of xenobiotics by cytochrome P450	5	0.072463768	0.0010045
hsa05204	Chemical carcinogenesis	5	0.064935065	0.001646987
hsa04726	Serotonergic synapse	5	0.044642857	0.008252113
hsa05322	Systemic lupus erythematosus	5	0.043103448	0.009530419
hsa04610	Complement and coagulation cascades	4	0.053333333	0.009773631
hsa00360	Phenylalanine metabolism	2	0.125	0.013470534
hsa04970	Salivary secretion	4	0.047058824	0.014971476
hsa04974	Protein digestion and absorption	4	0.046511628	0.01557085
hsa00140	Steroid hormone biosynthesis	3	0.055555556	0.022383946
hsa04750	Inflammatory mediator regulation of TRP channels	4	0.040816327	0.02395963
hsa00340	Histidine metabolism	2	0.090909091	0.024831356
hsa04921	Oxytocin signaling pathway	5	0.032894737	0.027571626
hsa04080	Neuroactive ligand-receptor interaction	7	0.025547445	0.033446169
hsa00830	Retinol metabolism	3	0.046875	0.034734996
hsa04919	Thyroid hormone signaling pathway	4	0.035714286	0.036665856
hsa04742	Taste transduction	3	0.044776119	0.039003143
hsa05031	Amphetamine addiction	3	0.044776119	0.039003143
hsa05214	Glioma	3	0.042857143	0.043526369
hsa00983	Drug metabolism - other enzymes	3	0.04109589	0.048300933
hsa05133	Pertussis	3	0.04109589	0.048300933
hsa04971	Gastric acid secretion	3	0.040540541	0.049947573
GO:0043271	negative regulation of ion transport	7	0.048275862	0.00050106
GO:1900371	regulation of purine nucleotide biosynthetic process	7	0.045454545	0.00071715
GO:0030808	regulation of nucleotide biosynthetic process	7	0.04516129	0.000745113
GO:0019373	epoxygenase P450 pathway	3	0.142857143	0.001014523
GO:0014065	phosphatidylinositol 3-kinase signaling	6	0.045801527	0.001655385
GO:0019369	arachidonic acid metabolic process	4	0.075471698	0.001656886
GO:0048015	phosphatidylinositol-mediated signaling	6	0.037735849	0.004342374
GO:2001257	regulation of cation channel activity	6	0.0375	0.004476564
GO:2000257	regulation of protein activation cascade	3	0.06122449	0.01155576
GO:0002544	chronic inflammatory response	2	0.105263158	0.014044865
GO:0052652	cyclic purine nucleotide metabolic process	4	0.038461538	0.01787916
GO:0008654	phospholipid biosynthetic process	7	0.024221453	0.021614065
GO:0046474	glycerophospholipid biosynthetic process	6	0.024489796	0.030932198

GO:0006171	cAMP biosynthetic process	3	0.041666667	0.031913732
GO:0030818	negative regulation of cAMP biosynthetic process	2	0.066666667	0.033361672
GO:0038111	interleukin-7-mediated signaling pathway	2	0.066666667	0.033361672
GO:0098761	cellular response to interleukin-7	2	0.064516129	0.035442037
GO:0071875	adrenergic receptor signaling pathway	2	0.0625	0.037571344
GO:0006644	phospholipid metabolic process	9	0.019067797	0.038471875
GO:0030814	regulation of cAMP metabolic process	3	0.037974684	0.040333433
GO:0050848	regulation of calcium-mediated signaling	3	0.037974684	0.040333433
GO:0007188	adenylate cyclase-modulating G-protein coupled receptor signaling pathway	5	0.025	0.043730329
GO:0009132	nucleoside diphosphate metabolic process	4	0.027777778	0.049994036
GO:0044344	cellular response to fibroblast growth factor stimulus	4	0.027777778	0.049994036

Supplementary Table 7. KEGG and GO enrichment analysis of 40 differentially expressed genes between NORA and CRA patients.

ID	Pathway_Name	Count	Enrichment	p_Value
hsa00564	Glycerophospholipid metabolism	2	0.020833333	0.007523158
hsa00604	Glycosphingolipid biosynthesis - ganglio series	1	0.071428571	0.01947223
hsa04964	Proximal tubule bicarbonate reclamation	1	0.043478261	0.031809201
GO:0050878	regulation of body fluid levels	5	0.01002004	0.001783292
GO:0048146	positive regulation of fibroblast proliferation	2	0.037735849	0.004040813
GO:0010001	glial cell differentiation	3	0.015228426	0.005161122
GO:0050891	multicellular organismal water homeostasis	2	0.03125	0.005839829
GO:0009247	glycolipid biosynthetic process	2	0.029411765	0.006569524
GO:0030104	water homeostasis	2	0.029411765	0.006569524
GO:0006643	membrane lipid metabolic process	3	0.013513514	0.00717923
GO:0050805	negative regulation of synaptic transmission	2	0.02739726	0.007536937
GO:0051702	interaction with symbiont	2	0.027027027	0.007737704
GO:2000027	regulation of organ morphogenesis	3	0.012048193	0.009820878
GO:0048145	regulation of fibroblast proliferation	2	0.022988506	0.010563645
GO:1902476	chloride transmembrane transport	2	0.022988506	0.010563645
GO:0048144	fibroblast proliferation	2	0.022727273	0.010797339
GO:0042063	gliogenesis	3	0.011538462	0.011039578
GO:0021782	glial cell development	2	0.021276596	0.012247256
GO:0052652	cyclic purine nucleotide metabolic process	2	0.019230769	0.014841957
GO:0009152	purine ribonucleotide biosynthetic process	3	0.010273973	0.015067585
GO:0009190	cyclic nucleotide biosynthetic process	2	0.019047619	0.015113421
GO:0098661	inorganic anion transmembrane transport	2	0.018867925	0.015387033
GO:0006164	purine nucleotide biosynthetic process	3	0.010135135	0.01562235
GO:0006821	chloride transport	2	0.018691589	0.015662786
GO:0060071	Wnt signaling pathway, planar cell polarity pathway	2	0.018181818	0.016502804
GO:0009260	ribonucleotide biosynthetic process	3	0.009836066	0.016912688
GO:0090175	regulation of establishment of planar polarity	2	0.017857143	0.017073371
GO:0072522	purine-containing compound biosynthetic process	3	0.00974026	0.0173558
GO:0046390	ribose phosphate biosynthetic process	3	0.009708738	0.017504952
GO:0019755	one-carbon compound transport	1	0.1	0.017769668

GO:0021670	lateral ventricle development	1	0.1	0.017769668
GO:0032429	regulation of phospholipase A2 activity	1	0.1	0.017769668
GO:0033131	regulation of glucokinase activity	1	0.1	0.017769668
GO:0060502	epithelial cell proliferation involved in lung morphogenesis	1	0.1	0.017769668
GO:0061140	lung secretory cell differentiation	1	0.1	0.017769668
GO:0106070	regulation of adenylate cyclase-activating G-protein coupled receptor signaling pathway	1	0.1	0.017769668
GO:0015791	polyol transport	1	0.090909091	0.019529734
GO:0046689	response to mercury ion	1	0.090909091	0.019529734
GO:0071474	cellular hyperosmotic response	1	0.090909091	0.019529734
GO:1902894	negative regulation of pri-miRNA transcription by RNA polymerase II	1	0.090909091	0.019529734
GO:1903299	regulation of hexokinase activity	1	0.090909091	0.019529734
GO:0001736	establishment of planar polarity	2	0.016393443	0.020050493
GO:0007164	establishment of tissue polarity	2	0.016393443	0.020050493
GO:0060456	positive regulation of digestive system process	1	0.083333333	0.021286748
GO:1900452	regulation of long term synaptic depression	1	0.083333333	0.021286748
GO:0006664	glycolipid metabolic process	2	0.015748031	0.021615076
GO:1903509	liposaccharide metabolic process	2	0.015748031	0.021615076
GO:0007596	blood coagulation	3	0.008797654	0.02266181
GO:0030157	pancreatic juice secretion	1	0.076923077	0.023040714
GO:0046415	urate metabolic process	1	0.076923077	0.023040714
GO:0046541	saliva secretion	1	0.076923077	0.023040714
GO:0050746	regulation of lipoprotein metabolic process	1	0.076923077	0.023040714
GO:0061029	eyelid development in camera-type eye	1	0.076923077	0.023040714
GO:0090179	planar cell polarity pathway involved in neural tube closure	1	0.076923077	0.023040714
GO:0090335	regulation of brown fat cell differentiation	1	0.076923077	0.023040714
GO:1900004	negative regulation of serine-type endopeptidase activity	1	0.076923077	0.023040714
GO:1902572	negative regulation of serine-type peptidase activity	1	0.076923077	0.023040714
GO:0007599	hemostasis	3	0.00867052	0.023535082
GO:0007586	digestion	2	0.015037594	0.023557703
GO:0050817	coagulation	3	0.008645533	0.02371193
GO:0030950	establishment or maintenance of actin cytoskeleton	1	0.071428571	0.024791638

	polarity			
GO:0042447	hormone catabolic process	1	0.071428571	0.024791638
GO:0072488	ammonium transmembrane transport	1	0.071428571	0.024791638
GO:0090178	regulation of establishment of planar polarity involved in neural tube closure	1	0.071428571	0.024791638
GO:0001738	morphogenesis of a polarized epithelium	2	0.01459854	0.024891501
GO:0090177	establishment of planar polarity involved in neural tube closure	1	0.066666667	0.026539526
GO:1900003	regulation of serine-type endopeptidase activity	1	0.066666667	0.026539526
GO:1902571	regulation of serine-type peptidase activity	1	0.066666667	0.026539526
GO:0035148	tube formation	2	0.013888889	0.027298545
GO:0030900	forebrain development	3	0.008174387	0.02740255
GO:0046467	membrane lipid biosynthetic process	2	0.013793103	0.027649865
GO:0009165	nucleotide biosynthetic process	3	0.008130081	0.027787703
GO:0060192	negative regulation of lipase activity	1	0.0625	0.028284381
GO:0071732	cellular response to nitric oxide	1	0.0625	0.028284381
GO:1901293	nucleoside phosphate biosynthetic process	3	0.008042895	0.028566778
GO:0009187	cyclic nucleotide metabolic process	2	0.013333333	0.029433947
GO:0002076	osteoblast development	1	0.058823529	0.03002621
GO:0042249	establishment of planar polarity of embryonic epithelium	1	0.058823529	0.03002621
GO:0043951	negative regulation of cAMP-mediated signaling	1	0.058823529	0.03002621
GO:0051262	protein tetramerization	2	0.013071895	0.030526126
GO:0035567	non-canonical Wnt signaling pathway	2	0.012987013	0.030893764
GO:1900371	regulation of purine nucleotide biosynthetic process	2	0.012987013	0.030893764
GO:0030808	regulation of nucleotide biosynthetic process	2	0.012903226	0.031263179
GO:0030952	establishment or maintenance of cytoskeleton polarity	1	0.055555556	0.031765017
GO:0070633	transepithelial transport	1	0.055555556	0.031765017
GO:1905276	regulation of epithelial tube formation	1	0.055555556	0.031765017
GO:0006497	protein lipidation	2	0.012578616	0.032758473
GO:0007517	muscle organ development	3	0.007575758	0.033272882
GO:0006833	water transport	1	0.052631579	0.033500807
GO:0015669	gas transport	1	0.052631579	0.033500807
GO:0033622	integrin activation	1	0.052631579	0.033500807

GO:0071731	response to nitric oxide	1	0.052631579	0.033500807
GO:1902170	cellular response to reactive nitrogen species	1	0.052631579	0.033500807
GO:0008361	regulation of cell size	2	0.012195122	0.034666733
GO:0042158	lipoprotein biosynthetic process	2	0.012195122	0.034666733
GO:0006665	sphingolipid metabolic process	2	0.012121212	0.035053535
GO:0014850	response to muscle activity	1	0.047619048	0.036963358
GO:0061436	establishment of skin barrier	1	0.047619048	0.036963358
GO:0097503	sialylation	1	0.047619048	0.036963358
GO:1901685	glutathione derivative metabolic process	1	0.047619048	0.036963358
GO:1901687	glutathione derivative biosynthetic process	1	0.047619048	0.036963358
GO:0006972	hyperosmotic response	1	0.045454545	0.038690128
GO:0007398	ectoderm development	1	0.045454545	0.038690128
GO:0009651	response to salt stress	1	0.045454545	0.038690128
GO:0042135	neurotransmitter catabolic process	1	0.045454545	0.038690128
GO:0060487	lung epithelial cell differentiation	1	0.045454545	0.038690128
GO:0001919	regulation of receptor recycling	1	0.043478261	0.040413902
GO:0006884	cell volume homeostasis	1	0.043478261	0.040413902
GO:0021952	central nervous system projection neuron axonogenesis	1	0.043478261	0.040413902
GO:0033561	regulation of water loss via skin	1	0.043478261	0.040413902
GO:0060479	lung cell differentiation	1	0.043478261	0.040413902
GO:0015698	inorganic anion transport	2	0.011111111	0.041055765
GO:1905330	regulation of morphogenesis of an epithelium	2	0.011049724	0.041468954
GO:0006688	glycosphingolipid biosynthetic process	1	0.041666667	0.042134685
GO:0021591	ventricular system development	1	0.041666667	0.042134685
GO:0032800	receptor biosynthetic process	1	0.041666667	0.042134685
GO:0071280	cellular response to copper ion	1	0.041666667	0.042134685
GO:0090103	cochlea morphogenesis	1	0.041666667	0.042134685
GO:0048839	inner ear development	2	0.010928962	0.042300101
GO:0051216	cartilage development	2	0.010928962	0.042300101
GO:0052547	regulation of peptidase activity	3	0.006802721	0.043586269
GO:0022011	myelination in peripheral nervous system	1	0.038461538	0.045567296
GO:0032292	peripheral nervous system axon ensheathment	1	0.038461538	0.045567296
GO:0042044	fluid transport	1	0.038461538	0.045567296
GO:0051346	negative regulation of hydrolase activity	3	0.00660793	0.046834206
GO:0006929	substrate-dependent cell migration	1	0.037037037	0.047279134
GO:1900542	regulation of purine nucleotide metabolic process	2	0.01025641	0.047417774

GO:0042157	lipoprotein metabolic process	2	0.01010101	0.0487314
GO:0031365	N-terminal protein amino acid modification	1	0.035714286	0.048988001
GO:0060292	long term synaptic depression	1	0.035714286	0.048988001
GO:0007188	adenylate cyclase-modulating G-protein coupled receptor signaling pathway	2	0.01	0.04961457
